# An artificial intelligence model to detect abnormal ejection fraction from non-contrast chest computed tomography: the CT–LVEF study

**DOI:** 10.1093/ehjdh/ztag088

**Published:** 2026-06-11

**Authors:** Jayant Raikhelkar, Zilong Bai, Ashley N Beecy, Ilan Richter, Fengbei Liu, Nusrat Binta Nizam, Varsha Kishore, Chris Kelsey, David vanMaanen, Jeffrey Ruhl, Naomi Tesfuzigta, Erica Lancet, Jay Leb, Alan Legasto, Pierre Elias, Timothy Poterucha, Deepa Kumaraiah, Martin Prince, Fei Wang, Gabriel Sayer, Deborah Estrin, Mert Sabuncu, Nir Uriel

**Affiliations:** Seymour, Paul, and Gloria Milstein Division of Cardiology, Department of Medicine, Columbia University Irving Medical Center/NewYork–Presbyterian Hospital, New York, NY, USA; Department of Population Health Sciences, Weill Cornell Medicine, Cornell University, NewYork, NY, USA; Sutter Health, Emeryville, CA, USA; Seymour, Paul, and Gloria Milstein Division of Cardiology, Department of Medicine, Columbia University Irving Medical Center/NewYork–Presbyterian Hospital, New York, NY, USA; Cornell Tech, NewYork, NY, USA; Cornell Tech, NewYork, NY, USA; Department of Computer Science, Cornell University, Ithaca, NY, USA; Information Technology Data Science, NewYork–Presbyterian Hospital, New York, NY, USA; Information Technology Data Science, NewYork–Presbyterian Hospital, New York, NY, USA; Information Technology Data Science, NewYork–Presbyterian Hospital, New York, NY, USA; Cornell Tech, NewYork, NY, USA; NewYork-Presbyterian Hospital, New York, NY, USA; Department of Radiology, Columbia University Irving Medical Center, NewYork, NY, USA; Department of Radiology, Weill Cornell Medicine, NewYork, NY, USA; Seymour, Paul, and Gloria Milstein Division of Cardiology, Department of Medicine, Columbia University Irving Medical Center/NewYork–Presbyterian Hospital, New York, NY, USA; Information Technology Data Science, NewYork–Presbyterian Hospital, New York, NY, USA; Departments of Biomedical Informatics, Columbia University, NewYork, NY, USA; Division of Cardiology, Mayo Clinic, Rochester, MN, USA; Seymour, Paul, and Gloria Milstein Division of Cardiology, Department of Medicine, Columbia University Irving Medical Center/NewYork–Presbyterian Hospital, New York, NY, USA; Division of Cardiology, Department of Medicine, Weil Cornell Medicine, NewYork, NY, USA; Department of Radiology, Weill Cornell Medicine, NewYork, NY, USA; Department of Population Health Sciences, Weill Cornell Medicine, Cornell University, NewYork, NY, USA; Seymour, Paul, and Gloria Milstein Division of Cardiology, Department of Medicine, Columbia University Irving Medical Center/NewYork–Presbyterian Hospital, New York, NY, USA; Department of Population Health Sciences, Weill Cornell Medicine, Cornell University, NewYork, NY, USA; Cornell Tech, NewYork, NY, USA; Department of Computer Science, Cornell University, Ithaca, NY, USA; Cornell Tech, NewYork, NY, USA; School of Electrical and Computer Engineering, Cornell University, Ithaca, NY, USA; Department of Radiology, Weill Cornell Medical School, NewYork, NY, USA; Seymour, Paul, and Gloria Milstein Division of Cardiology, Department of Medicine, Columbia University Irving Medical Center/NewYork–Presbyterian Hospital, New York, NY, USA

**Keywords:** Heart Failure, Artificial Intelligence, Opportunistic Screening

## Abstract

**Aims:**

Heart failure (HF), a major global health challenge, affects millions worldwide and poses substantial healthcare and economic burdens. It is estimated that a large proportion of those with early systolic dysfunction remain asymptomatic at a stage when guideline-directed medical therapies have been shown to prevent disease progression. To develop an artificial intelligence (AI) model capable of predicting abnormal left ventricular ejection fraction (EF) directly from static, non-gated, non-contrast chest computed tomography (CT) scans as a form of opportunistic screening,

**Methods and results:**

Using a multi-institutional dataset of 34 058 paired non- contrast CT images and echocardiogram reports from two academic centres, we trained our model of classification for predicting left-ventricle ejection fraction (LVEF) categories: abnormal EF (EF < 50%) vs. normal on 25 948 studies. We validated the model on 8110 paired chest CT and echocardiogram results from a separate institution. The model achieved an area under the receiver operating characteristic (AUROC) curve of 0.786 on the hold-out test set and 0.762 on external validation to detect an abnormal EF (<50%). Beyond strong predictive performance, the AI model surpassed expert radiologists in both accuracy and efficiency and provided interpretable visualizations highlighting imaging features linked to reduced LVEF.

**Conclusion:**

In this study, we developed and validated an AI model capable of predicting abnormal LVEF directly from static, non-gated, non-contrast chest CT scans, a novel application for an imaging modality typically used for unrelated indications as a form of opportunistic screening. This technology holds significant promise for early detection of systolic HF, reducing the diagnostic gap, and improving outcomes in asymptomatic HF patients.

## Graphical Abstract

**Graphical Abstract:**
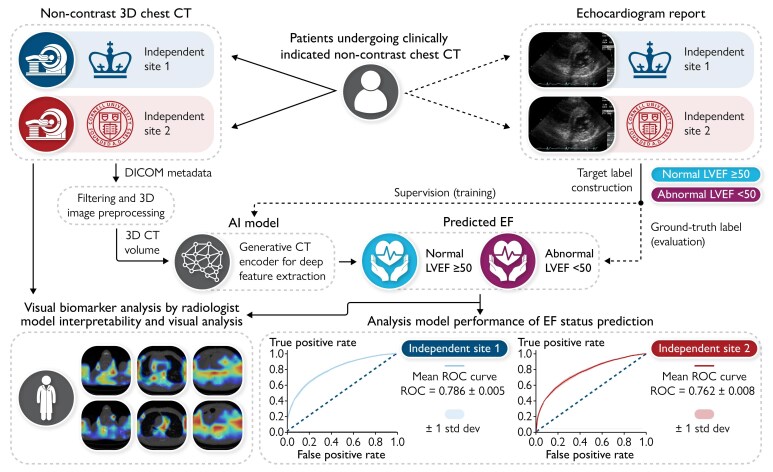

## Introduction

Heart Failure (HF) has been declared a global pandemic with over 64 million individuals afflicted worldwide.^1^ About 6.7 million of these individuals are in the USA, and this number is expected to rise to 8.5 million by 2030.^1,2^ Despite improvements in medical and surgical treatment, HF remains a leading cause of hospitalizations and mortality.^3^ In addition, the worldwide economic burden of HF is estimated to be $346 billion US dollars annually.^4^

The left ventricular ejection fraction (EF) is a critical parameter which is used to phenotype HF into several types, namely HF with reduced EF (HFrEF), HF with mid-range EF (HFmrEF), and HF with preserved EF (HFpEF).^5^ There is evidence for early initiation of lifestyle modifications and medications that constitute guideline-directed medical therapy (GDMT) for both HfrEF and HFmrHF to decrease both HF hospitalizations and mortality.^6–8^ It is estimated that a substantial number of patients remain undiagnosed with HfmrEF and HfrEF and thus are unable to be started on appropriate HF therapies.^2^ There is an unmet need for earlier detection of these patients with the goal of improving outcomes by means of initiation of lifestyle modification and GDMT at an earlier stage of disease.

Computed tomography (CT) scans are a common medical imaging modality, with over 80 million studies being performed yearly in the USA.^9^ Chest CT scans are frequently performed in both inpatient and outpatient settings for a wide range of indications, such as screening for lung cancer, acute pulmonary embolism detection, and evaluation for infectious diseases like pneumonia and trauma. These studies contain a wealth of biometric information, and several studies have recently investigated the use of artificial intelligence (AI) based automated algorithms applied to CT scans for identifying patients at higher risk for adverse events, including cardiovascular adverse events.^10,11^

In this study, we aimed to develop an AI model capable of predicting abnormal left-ventricle ejection fraction (LVEF), a dynamic physiological parameter, directly from non-gated, non-contrast chest CT scans.

## Methods

### Ethics approval

This study was approved by the institutional review boards at Weill Cornell Medicine and Columbia University Irving Medical Center. A waiver for informed consent was obtained.

### Data curation and preprocessing

**Figure 1:**
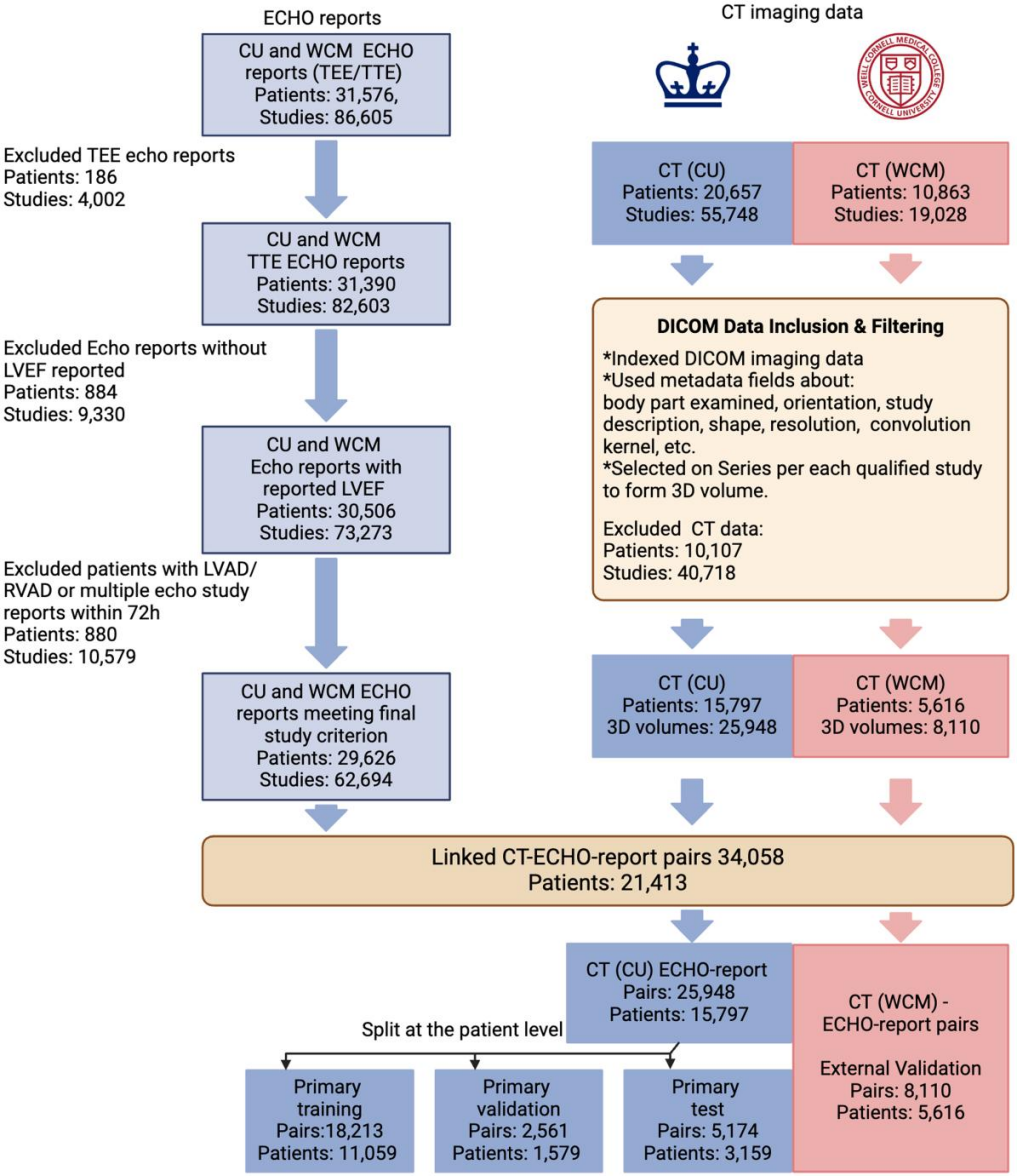
Data curation and preprocessing flowchart.

All patients aged 18 years or older who had at least one echocardiogram (echo) within 6 months before or after a non-contrast chest CT at either Columbia University Irving Medical Center (CU) or Weill Cornell Medical Center (WCM) between July 2005 and August 2023 were identified. This initial data collection process identified 31 576 patients. The echo study reports and CT imaging data were then filtered, linked, and preprocessed according to the protocols detailed in the following sections. Details of convolutional kernels extracted from DICOM metadata across cohorts are provided in Supplementary material online, *Table S1*. Eventually, we identified a cohort of 19 410 patients with a total of 34 058 qualified echo-CT data pairs. From this cohort, two study groups were formed based on the source of the CT data: CU (14 083 unique patients with 25 948 echo-CT pairs) and WCM (5327 unique patients with 8110 echo-CT pairs). The CU group served as the primary dataset for AI model development, while the WCM group was reserved for external validation. The CU dataset was further randomly split at the patient level into training, validation, and test subsets (i.e. each patient was assigned to only one of these three subsets). Specifically, the CU primary test subset in *Figure 1* is referred to as the CU hold-out test set as it is reserved purely for performance evaluation, ensuring no data leakage into model training. For our AI models, we created CT–echo study pairs using a CT volume image as the input and the LVEF value derived from an echo report as the target label for each pair. These pairs are formed and curated through *data linkage*, *data filtering*, and the *3D CT volume preprocessing* (see Supplementary Materials for details of each stage). The data curation and preprocessing flowchart is in *Figure 1*.

#### 3D non-contrast chest computed tomography volume preprocessing

DICOM images of CT scans in Hounsfield Units (HU) were processed by a function where we rescaled slope and intercept to provide a consistent and standard measurement for data interpretation and analysis. The 2D DICOM images of the same CT series were merged into a 3D Numpy array to represent this 3D CT volume. A window of [−1,000, 1000] was applied to the intensity values to exclude air (<−1000 HU) and bones (>1000 HU). Further steps based on OTSU thresholding were taken to focus on the patient's body region (see Supplementary Materials for details). The interpolation function from the Python library Scipy ndimage^12^ was used to rescale the 3D voxel spacing of all the 3D CT volumes into a standardized resolution of $2\times 2\times 2\;mm^{3}$, where the spline interpolation was applied. Finally, 3D cropping and zero padding were applied where applicable, to resize the 3D volume into a volume of size 164 × 164 × 164.

### AI model: classifier based on a pretrained computed tomography–vision transformer encoder

**Figure 2:**
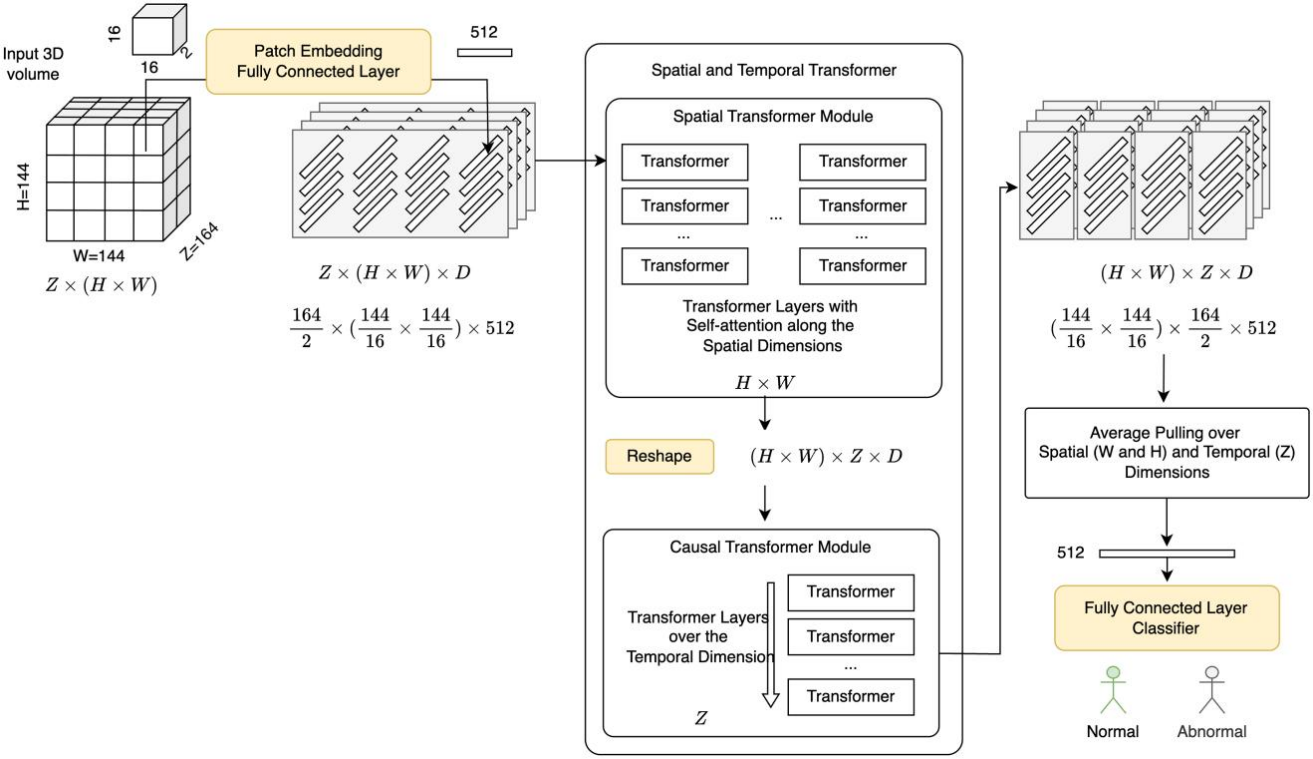
Overview of the vision transformer-based artificial intelligence model architecture.

We developed a classifier for 3D CT images by adapting a pretrained vision transformer (ViT) architecture,^13^ using the encoder of the CT–ViT framework (GenerateCT^14^) as the backbone (*Figure 2*). The encoder processes a pre-processed 3D CT volume of 164 × 164 × 164, randomly cropped to 164×144×144 during training, and outputs a 512-dimensional feature vector, which is passed to a fully connected classifier to generate a probability of low LVEF. The pretrained weights were fine-tuned on our CU training cohort. The model was optimized using Binary Cross Entropy loss with AdamW at learning rate 1 × 10^−5^, weight decay 1 × 10^−4^, batch size 8 for 20 epochs, with learning rate halved at epoch 15. Data augmentation included random cropping and horizontal flipping; centre-cropping was used at test time. To characterize the model’s behaviour and reliability, we repeated training five times using different random seeds. All reported performance metrics are presented as mean ± standard deviation (95% confidence interval) across these five runs unless otherwise specified. All experiments were conducted on an NVIDIA A100 GPU. Additional architectural details of the CT–ViT encoder, patch embedding layer, spatial/causal transformer modules, and the mathematical formulation of the loss function are provided in the Supplementary material.

### Baseline model using demographic and computed tomography report-derived clinical features

We implemented baseline models using both a RF classifier and a gradient boosting model (XGBoost) trained on demographic features (age, sex, and race) and clinical information extracted from CT reports. Clinical information was represented by 260 binary features corresponding to distinct ICD-9 diagnosis codes present across the CU and WCM cohorts. Each CT report was encoded as a 260-dimensional binary vector indicating the presence or absence of each ICD-9 code, which was concatenated with the demographic features to form the model input.

The RF model was trained using standard ensemble settings, while the XGBoost model was trained using gradient boosting decision trees with regularization to optimize predictive performance on tabular data. Both models were trained and evaluated using the same training, validation, and test splits as the AI model.

### Evaluation metrics

#### Left-ventricle ejection fraction-based classification metric

LVEF values were collected from the echo report to form labels for both training and testing. All EF values were derived via 2D echocardiography by means of a waterfall logic (see Supplementary material online, *Table S2*). They were binarized according to a clinician-determined threshold (50%), where higher than or equal to this threshold is considered normal and lower than this threshold is considered to be an indication of reduced ejection fraction (HFmrEF and/or HFrEF) or LV systolic dysfunction. A CT–echo pair where the echo-derived LVEF <50% was defined as the ‘positive’ class for calculation of receiver operating characteristic (ROC), area under the receiver operating characteristic (AUROC), sensitivity, specificity, accuracy, and balanced accuracy.

#### Calibration assessment

In addition to discrimination metrics, we evaluated model calibration to assess the agreement between predicted probabilities and observed outcomes. Calibration was quantified using the Brier score, defined as the mean squared difference between predicted probabilities and ground-truth labels. Calibration curves (reliability plots) were generated by grouping predictions into bins and comparing average predicted probabilities with observed event rates. To account for variability in model training, Brier scores were computed across models trained with different random seeds, and mean values with standard deviations were reported.

### Interpretation of the model

#### Visualization of contributive regions of artificial intelligence model

To understand the predictions made by the deep learning model’s predictions, we used Gradient-weighted Class Activation Mapping (Grad-CAM),^15^ a technique for visualizing the regions within an input image that contributes most significantly to the model's output prediction. Given that our AI model is a specialized ViT architecture with separate self-attention blocks for spatial and slice-wise (*z*-axis) processing, we generated two distinct saliency maps for each CT volume. The spatial saliency map is derived from the first four self-attention blocks, which operate on the axial plane (W×H), while the Z-dimension saliency map is derived from the second four self-attention blocks, which attend to the importance of individual slices along the vertical axis. These maps help localize both spatially salient regions on each slice and identify the most relevant slices within the volume.

**Figure 3:**
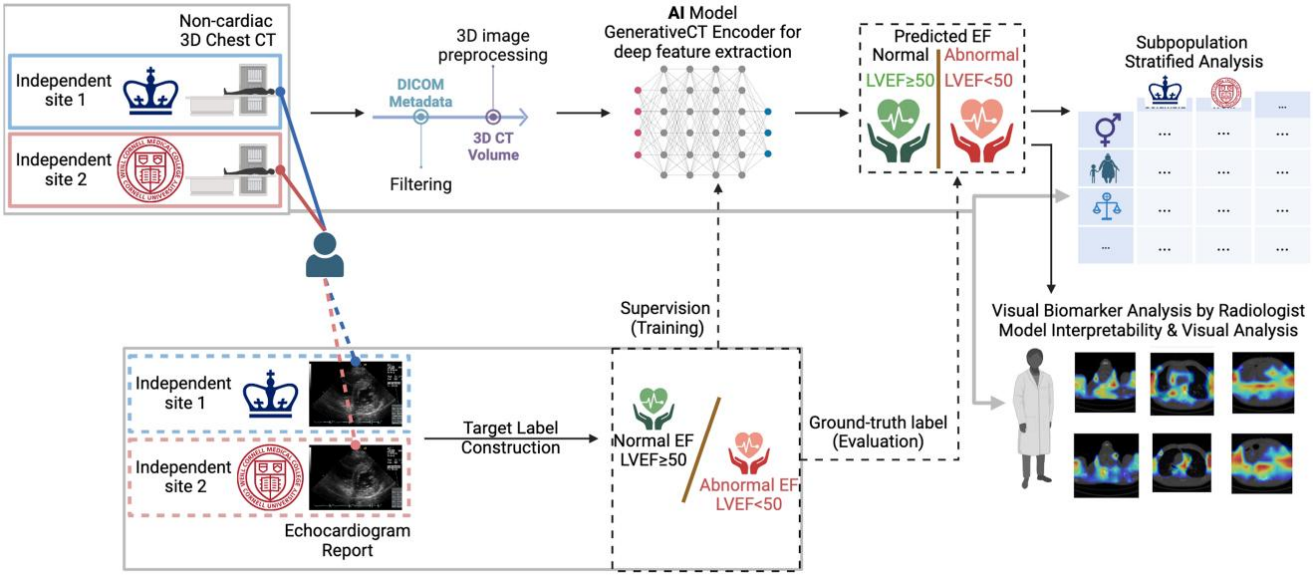
Study design. We collected computed tomography imaging data and echo reports from two independent sites, Columbia University Irving Medical Center and Weill Cornell Medicine. The computed tomography and echo studies were matched per patient based on specific inclusion and exclusion criteria to create input-label pairs for developing our artificial intelligence model. Preprocessed computed tomography scans served as model inputs, while left-ventricle ejection fraction values from echo studies provided binary labels for training and ground-truth performance evaluation. We conducted sub-cohort evaluations by stratifying the patient cohort based on demographic attributes and varying time intervals between computed tomography and echo studies to explore different application scenarios. Additionally, a radiologist reviewed randomly selected computed tomography scans without being informed of the model's prediction results.

A summary of the methodology is visualized in *Figure 3*.

#### Comparison with radiologist manual analysis and inference

We compared LVEF status predictions made by our AI model against those made by dedicated thoracic radiologists using CT imaging data to benchmark performance and identify strengths and weaknesses. Specifically, we randomly selected 90 non-contrast CT scans from the CU hold-out test set and 100 from the WCM external validation set, with similar rates of abnormal EF status as their respective full test sets. LVEF status information was withheld before sending the patient lists to the radiologists to ensure unbiased manual interpretation.

The manual prediction process was done by two highly experienced thoracic radiologists, one (RAD1) from CU and the other (RAD2) from WCM. Both radiologists independently reviewed the CU 90 CT scans and made predictions regarding LVEF status. RAD2 also reviewed the 100 WCM scans. Using the LVEF status derived from echo reports as ground truth, our AI model was more accurate in determination of abnormal EF. The radiologists used qualitative visual assessment for prediction of abnormal EF.

### Validation on independent consecutive test sets

To further evaluate the performance of our developed AI model in a real-world clinical setting, we constructed another two independent testing sets using the CT studies and echo reports collected from subjects admitted to CU (August to December 2023) and WCM (August 2023 to July 2024) of the NYP healthcare systems. Their CT and echo data were collected after the primary (retrospective) dataset, ensuring temporal separation from the training data.

### Decision curve analysis of the artificial intelligence model

To assess the potential clinical utility of the AI model for screening abnormal LVEF, we performed decision-curve analysis. Net benefit was calculated across a range of threshold probabilities and compared with two baseline strategies: treating all patients as having abnormal LVEF and treating none. Decision curves were generated for each cohort using predicted probabilities from the AI model. For models trained with different random seeds, the mean net benefit and standard deviation across five runs were reported at each threshold probability.

Compliance with the PRIME 2.0 checklist is summarized in Supplementary material online, *Table S9*.

## Results

### Curated and preprocessed dataset

The curated dataset covered diverse demographics, with a balanced gender distribution. *Table 1* summarizes statistics for study pairs stratified by the institution of CT data collection and patient demographics: genders (male and female), age groups (18–40, 40–65, and 65–120 years), and racial groups [Asian/Native Hawaiian/Other Pacific Islands (ANO), White, and Black or African American (BAA)]. A consolidated comparison of baseline characteristics across the CU training, validation, and hold-out test sets and the WCM external validation cohort is provided in Supplementary material online, *Table S3*. *Table 2* presents the summary statistics for the critical acquisition parameters, kVp, tube current, and slice thickness, with statistical comparison between the normal and abnormal EF subgroups, stratified by institution and patient demographics. *Table 3* presents the top three most frequently mentioned diagnosis codes in the ‘CLINICAL INFORMATION’ field of the CT reports in each institution and patient demographics stratified subset. *Table 4* presents the distribution of CT–echo time intervals varied across cohorts. The median CT–echo interval ranged from 12.7 to 24.2 days across retrospective cohorts and from 16.8 to 23.0 days in prospective cohorts. A substantial proportion of CT–echo pairs were temporally proximate, with 11.8–20.7% occurring within 1 day, 32.0–42.1% within 1 week, and 53.7–62.4% within 1 month. These distributions support subsequent analyses examining model performance as a function of CT–echo temporal proximity.

**Table 1:** Population description

	Columbia University Irving Medical Center Total: 25 948 chest CTs		Weill Cornell Medical College Total: 8110 chest CTs		Between-site Chi-square test of independence *P*-value		
	Normal (LVEF ≥ 50)—21 787 (83.96%)	Abnormal (LVEF < 50)—4161 (16.04%)	Normal (LVEF ≥ 50)—6695 (82.70%)	Abnormal (LVEF < 50)—1415 (17.29%)	Overall	Normal	Abnormal
Patient sex*							
Male	10 351 (47.51%)	2616 (62.87%)	3207 (47.90%)	942 (66.57%)	<0.0001	<0.0001	0.5784
Female	9799 (44.98%)	1278 (30.71%)	3486 (52.07%)	472 (33.36%)			
Patient age (years)							
18–39	2011 (9.23%)	218 (5.23%)	351 (5.24%)	56 (3.96%)	<0.0001	<0.0001	0.0102
40–64	8276 (37.99%)	1249 (30.02%)	2204 (32.92%)	387 (27.35%)			
65 and above	11 500 (52.78%)	2694 (64.74%)	4140 (61.84%)	972 (68.69%)			
Patient race*							
Asian/Native Hawaiian/Other Pacific Islander	784 (3.60%)	90 (2.16%)	403 (6.02%)	90 (6.36%)	<0.0001	<0.0001	<0.0001
White	10 761 (49.39%)	1754 (42.15%)	3596 (53.71%)	699 (42.33%)			
Black or African American	3140 (14.41%)	654 (15.72%)	897 (13.40%)	212 (14.98%)			

The data sets analysed are compiled from Columbia University Irving Medical Center (CU) and Weill Cornell Medicine (WCM). Each instance in the study consists of an input-label pair, where the input is preprocessed non-contrast chest computed tomography (CT) volumetric data, and the label reflects normal or abnormal left ventricle ejection fraction (LVEF) status derived from the associated echocardiography report. The AI model is trained on 80% of the instances with CT images from the Columbia dataset. Subpopulation statistics are calculated by stratifying the data according to the sites where the CT studies were collected and the demographics of the patients. The numbers indicate the pairs with CTs from different sites. One patient may be associated with different LVEF statuses by different CTs. *Percentages for Patient Sex or Race subgroups do not sum to 100% due to additional categories with insufficient sample sizes. These categories, labeled as ‘Others’ or ‘Not provided,’ were omitted from the population statistics for clarity. Chi-square test of independence was conducted to compare the distributional differences between CU and WCM in terms of patient sex, age groups, and race. The analyses were performed on all patients as well as the normal and abnormal subgroups for each variable.

**Table 2:** Summary statistics and statistical comparisons of important acquisition parameters

	Columbia University Irving Medical Center			Weill Cornell Medical College		
	kVp	TC	ST	kVp	TC	ST
All	119.8 ± 3.5 *P* = 0.1821	256.8 ± 164.9 *P* = 0.8643	1.154 ± 0.27 *P = 0.023	103.2 ± 8.2 *P = 0.0257	274.7 ± 131.1 **P = 0.0074	1.008 ± 0.30 *P = 0.0303
Patient sex						
Male	119.7 ± 3.7 *P* = 0.0392	278.7 ± 166.8 *P* = 0.0802	1.143 ± 0.28 *P* = 0.4501	103.1 ± 7.8 *P* = 0.1138	292.3 ± 132.5 *P* = 0.1347	1.006 ± 0.30 *P* = 0.0637
Female	119.9 ± 3.4 *P* = 0.3755	235.7 ± 160.9 *P = 0.0175	1.165 ± 0.26 *P* = 0.2553	103.3 ± 8.6 *P* = 0.1646	256.1 ± 127.0 *P* = 0.6412	1.009 ± 0.30 *P* = 0.1898
Patient age						
18–39	119.7 ± 4.0 *P* = 0.332	218.7 ± 156.0 *P = 0.0251	1.166 ± 0.25 *P* = 0.7846	102.4 ± 8.0 *P* = 0.5415	267.4 ± 134.7 *P* = 0.1002	1.006 ± 0.30 *P* = 0.1595
40–64	119.8 ± 3.5 *P* = 0.6527	270.1 ± 169.8 *P* = 0.246	1.155 ± 0.27 ***P = 0.0004	103.4 ± 8.3 *P* = 0.5067	283.5 ± 133.4 **P = 0.0021	0.997 ± 0.31 *P = 0.0112
65 and above	119.8 ± 3.4 *P* = 0.0866	253.7 ± 161.8 *P* = 0.8142	1.151 ± 0.27 *P* = 0.9896	103.2 ± 8.2 *P = 0.0289	269.9 ± 129.2 *P* = 0.6826	1.015 ± 0.30 *P* = 0.3746
Patient race						
ANO	120.0 ± 3.3 *P* = 0.2151	201.2 ± 142.2 *P* = 0.1818	1.161 ± 0.23 *P* = 0.1747	101.9 ± 8.1 *P* = 0.129	233.4 ± 106.8 ***P = 0.0001	1.023 ± 0.30 *P* = 0.0686
White	119.7 ± 3.6 *P* = 0.0988	256.5 ± 167.2 *P = 0.0265	1.146 ± 0.26 ***P = 0.0006	103.0 ± 8.0 *P* = 0.1723	276.1 ± 130.7 *P* = 0.1379	1.013 ± 0.30 *P* = 0.3196
BAA	120.0 ± 2.9 *P* = 0.7273	259.3 ± 170.2 *P* = 0.0657	1.157 ± 0.25 *P* = 0.5167	103.8 ± 8.5 **P = 0.0022	300.9 ± 142.1 *P* = 0.8752	1.015 ± 0.30 *P* = 0.6968

The mean and standard deviation of kVp, tube current (TC) and slice thickness (ST) collected at the central slice of each computed tomography (CT) imaging data of our curated datasets from Columbia University Irving Medical Center (CU) and Weill Cornell Medical Center (WCM), respectively and stratified by demographics. In each subset, the mean ± standard deviation of the acquisition parameters and the *P*-value from the independent two-sample test (i.e. the Welch’s test) for comparison between positive and negative groups in this subset were reported (*P < 0.05, **P < 0.01, and ***P < 0.001).

For abbreviations in patient race, ANO, Asian/Native Hawaiian/Other Pacific Islander; BAA, Black or African American.

**Table 3:** Summary of diagnostic information

	Columbia University Irving Medical Center	Weill Cornell Medical College
All	513.0—Abscess of lung, 996.84—Complications of transplanted lung, 786.2—Cough	513.0—Abscess of lung, 996.84—Complications of transplanted lung, 786.2—Cough
Patient sex		
Male	513.0—Abscess of lung, 996.84—Complications of transplanted lung, 786.2—Cough	513.0—Abscess of lung, 786.2—Cough, 996.84—Complications of transplanted lung
Female	996.84—Complications of transplanted lung, 513.0—Abscess of lung, 786.2—Cough	786.2—Cough, 513.0—Abscess of lung, 996.84—Complications of transplanted lung
Patient age		
18–39	996.84—Complications of transplanted lung, 513.0—Abscess of lung, 786.2—Cough	513.0—Abscess of lung, 996.84—Complications of transplanted lung, 786.2—Cough
40–64	996.84—Complications of transplanted lung, 513.0—Abscess of lung, 786.2—Cough	513.0—Abscess of lung, 786.2—Cough, 996.84—Complications of transplanted lung
65 and above	786.05—Shortness of breath, 786.2—Cough, 513.0—Abscess of lung	786.2—Cough, 513.0—Abscess of lung, 786.05—Shortness of breath
Patient race		
ANO	513.0—Abscess of lung, 996.84—Complications of transplanted lung, 572.3—Portal hypertension	518.89—Other diseases of lung, not elsewhere classified, 786.2:—Cough, 162.9—Malignant neoplasm of bronchus and lung, unspecified
White	996.84—Complications of transplanted lung, 786.2—Cough, 786.05—Shortness of breath	996.84—Complications of transplanted lung, 786.2—Cough, 513.0—Abscess of lung
BAA	513.0—Abscess of lung, 786.2—Cough, 786.05—Shortness of breath	786.2—Cough, 786.05—Shortness of breath, 996.84—Complications of transplanted lung

The top 3 most frequently mentioned ICD-9 diagnosis codes extracted from the CLINICAL INFORMATION field of CT reports, stratified by institution (CU and WCM) and patient demographics (age, gender, race).

ANO, Asian/Native Hawaiian/Other Pacific Islander; BAA, Black or African American; CT, computed tomography; CU, Columbia University Irving Medical Center; WCM, Weill Cornell Medical Center.

**Table 4:** Distribution of absolute time differences between CT and echocardiography studies across cohorts

Cohort	*N*	Median (days)	IQR (days)	≤1 day (%)	≤7 days (%)	≤30 days (%)
CU primary test	5174	12.7	1.8–62.0	20.7	42.1	62.4
WCM external validation	8110	24.2	3.9–78.0	12.5	32.0	53.7
CU independent consecutive test	1411	16.8	2.0–79.0	18.4	38.3	57.9
WCM independent consecutive test	817	23.0	3.8–84.1	11.8	32.4	53.9

CU, Columbia University Irving Medical Center; WCM, Weill Cornell Medical Center.

### AI model evaluation

**Figure 4:**
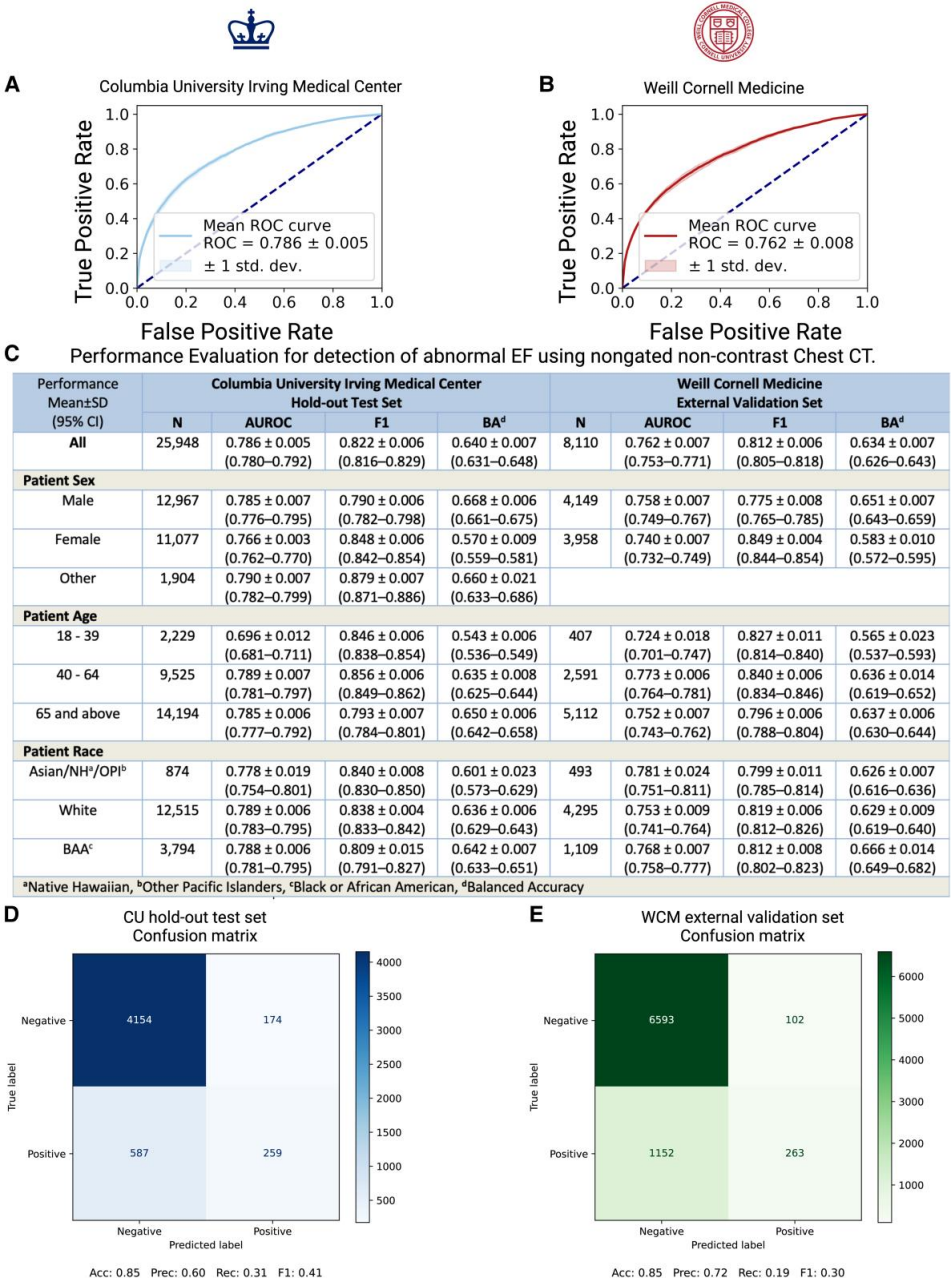
Performance evaluation: detection of abnormal ejection fraction using non-gated non-contrast chest computed tomography and (*B*) receiver operating characteristic curves and area under the receiver operating characteristic scores for site-specific test set performance: for Columbia University Irving Medical Center (*A*) and Weill Cornell Medical Center (*B*), respectively. (*C*) Average performance metrics (in area under the receiver operating characteristic, F1-score, and Balanced Accuracy) for subpopulations stratified from the test set according to site and demographic attributes: gender, age, and race. (*D* and *E*) Confusion matrices for Columbia University Irving Medical Center hold-out test set (*D*) and Weill Cornell Medical Center external validation set (*E*), respectively. See *Table 1* for corresponding population statistics.

On the CU hold-out test set, the AI model achieved an AUROC of 0.786 ± 0.005 [95% confidence interval (CI): 0.780–0.792) and an F1 score of 0.822 ± 0.005 (95% CI: 0.816–0.829) for predicting LVEF-derived status labels (*n* = 5,174, 20%) (*Figure 4*). We investigated the sensitivity of our model given various levels of specificity and vice versa on both the CU hold-out test set (see Supplementary material online, *Table S4*). Performance metrics were further analysed across demographic subgroups (Table c in *Figure 4*). Model performance at multi-class formulations (e.g. 3-class and 4-class by introducing multiple thresholds of LVEF) and additional clinically relevant LVEF thresholds (e.g. 40% and 35%) are provided in Supplementary *Tables 5* and *6*, respectively. The number of instances in each demographic subgroup is summarized in *Table 1* and annotated to Table c in *Figure 4*. For each demographic attribute, our model performed best within the male subgroup with an AUROC of 0.785 ± 0.008 (95% CI: 0.776–0.795), within the senior (65∼120 years) age subgroup with an AUROC of 0.785 ± 0.006 (95% CI: 0.777–0.792), and within the ANO racial subgroup with an AUROC of 0.778 ± 0.019 (95% CI: 0.754–0.801) (Table c in *Figure 4*). We conducted the permutation-based one-way ANOVA test to assess the statistical significance of model performance differences across demographic strata. The performance demonstrated statistical significance across different patient sex subgroups (*P*-value = 0.0018) and across different patient age subgroups (*P*-value = 0.0008). The performance did not show a statistically significant difference across different race subgroups (*P*-value = 0.4463). For each demographic subgroup, we further investigated the performance of our AI model with respect to LVEF measurements from different CT-to-echo study time intervals: 1 day, 1 week, and 1 month. We considered non-directional time intervals, meaning that both CT-before-echo and echo-before-CT pairs were eligible, provided that the absolute time difference between the two studies met the predefined stratification criteria. Performance stratified by temporal directionality (CT before echocardiogram vs. echocardiogram before CT) is provided in Supplementary material online, *Table S7*. For the overall CU hold-out test set, AUROC was higher for the ≤1-day interval subset than for the full cohort (0.800 vs. 0.786; ΔAUROC = 0.014; Holm-adjusted *P* = 0.004), and the ≤1-month interval subset was also higher than the full cohort (0.794 vs. 0.786; ΔAUROC = 0.008; Holm-adjusted *P* = 0.049), whereas the ≤1-week interval subset was not significantly different from the full cohort (0.788 vs. 0.786; Holm-adjusted *P* = 0.506). The ≤1-day interval subset also had a higher AUROC than the ≤1-week and ≤1-month subsets in the overall CU cohort (Holm-adjusted *P* = 0.003 and *P* = 0.045, respectively). However, this pattern was not uniform across demographic subgroups; the highest AUROC occurred at ≤1 day in some subgroups but at longer intervals in others (*Table 5*). These findings suggest that CT–echo temporal proximity may influence model performance, but subgroup-specific interval trends should be interpreted as exploratory.

**Table 5:** Performance evaluation in subgroups stratified by patient demographics and time interval between CT and echo studies

Performance Mean ± SD (95% CI)	Columbia University Irving Medical Center Hold-out test set (AUROC)				Weill Cornell Medicine external validation set (AUROC)			
	All	1 day	1 week	1 month	All	1 day	1 week	1 month
All	0.786 ± 0.005 (0.780–0.792)	0.800 ± 0.004 (0.795–0.805)	0.788 ± 0.004 (0.783–0.793)	0.794 ± 0.004 (0.789–0.799)	0.762 ± 0.007 (0.753–0.771)	0.782 ± 0.007 (0.773–0.791)	0.769 ± 0.006 (0.761–0.777)	0.788 ± 0.008 (0.779–0.798)
Patients sex								
Male	0.785 ± 0.008 (0.776–0.795)	0.799 ± 0.006 (0.791–0.806)	0.786 ± 0.007 (0.777–0.795)	0.795 ± 0.008 (0.786–0.805)	0.758 ± 0.007 (0.749–0.767)	0.781 ± 0.009 (0.770–0.792)	0.770 ± 0.006 (0.762–0.779)	0.785 ± 0.010 (0.773–0.798)
Female	0.766 ± 0.003 (0.762–0.770)	0.788 ± 0.003 (0.784–0.792)	0.772 ± 0.004 (0.767–0.778)	0.781 ± 0.005 (0.774–0.787)	0.740 ± 0.007 (0.732–0.749)	0.755 ± 0.006 (0.747–0.763)	0.740 ± 0.007 (0.731–0.749)	0.768 ± 0.008 (0.758–0.778)
Other	0.790 ± 0.007 (0.782–0.799)	0.790 ± 0.010 (0.777–0.802)	0.791 ± 0.008 (0.781–0.800)	0.769 ± 0.008 (0.759–0.779)	N/A			
Patients age								
18–39	0.696 ± 0.012 (0.681–0.711)	0.688 ± 0.011 (0.673–0.702)	0.681 ± 0.008 (0.670–0.691)	0.691 ± 0.013 (0.674–0.708)	0.724 ± 0.018 (0.701–0.747)	0.724 ± 0.020 (0.698–0.750)	0.725 ± 0.018 (0.702–0.749)	0.735 ± 0.025 (0.703–0.767)
40–64	0.789 ± 0.007 (0.781–0.797)	0.809 ± 0.005 (0.802–0.815)	0.794 ± 0.005 (0.788–0.800)	0.797 ± 0.004 (0.792–0.802)	0.773 ± 0.006 (0.764–0.781)	0.773 ± 0.008 (0.762–0.784)	0.777 ± 0.005 (0.770–0.784)	0.807 ± 0.008 (0.797–0.818)
65 and above	0.785 ± 0.006 (0.777–0.792)	0.809 ± 0.005 (0.802–0.815)	0.794 ± 0.005 (0.788–0.800)	0.790 ± 0.004 (0.785–0.794)	0.752 ± 0.008 (0.743–0.762)	0.773 ± 0.007 (0.763–0.783)	0.764 ± 0.007 (0.755–0.773)	0.778 ± 0.007 (0.769–0.787)
Race								
Asian/NH^a^/OPI^b^	0.778 ± 0.019 (0.754–0.801)	0.790 ± 0.021 (0.763–0.816)	0.787 ± 0.018 (0.765–0.809)	0.806 ± 0.024 (0.775–0.836)	0.781 ± 0.024 (0.751–0.811)	0.824 ± 0.022 (0.796–0.852)	0.802 ± 0.020 (0.775–0.829)	0.837 ± 0.028 (0.802–0.872)
White	0.789 ± 0.006 (0.783–0.795)	0.802 ± 0.004 (0.797–0.807)	0.783 ± 0.004 (0.778–0.789)	0.791 ± 0.005 (0.785–0.797)	0.753 ± 0.009 (0.741–0.764)	0.767 ± 0.010 (0.754–0.780)	0.759 ± 0.010 (0.746–0.772)	0.774 ± 0.011 (0.760–0.788)
BAA^c^	0.788 ± 0.006 (0.781–0.795)	0.804 ± 0.008 (0.793–0.815)	0.789 ± 0.009 (0.778–0.801)	0.813 ± 0.008 (0.802–0.823)	0.768 ± 0.007 (0.758–0.777)	0.790 ± 0.005 (0.783–0.798)	0.780 ± 0.006 (0.771–0.788)	0.797 ± 0.009 (0.785–0.809)

AUROC, area under the receiver operating characteristic.

^a^Native Hawaiian.

^b^Other Pacific Islanders.

^c^Black or African American.

**Table 6:** Baseline model performance evaluation

Model	Evaluation dataset	AUROC	Balanced Accuracy	F1 score
Random Forest	CUMC hold-out test set	0.538 ± 0.017 (0.517–0.559)	0.506 ± 0.007 (0.497–0.515)	0.107 ± 0.018 (0.086–0.131)
XGBoost	CUMC hold-out test set	0.607 ± 0.010 (0.588–0.625)	0.580 ± 0.011 (0.563–0.596)	0.309 ± 0.009 (0.289–0.330)
Random Forest	WCM external validation set	0.550 ± 0.013 (0.533–0.565)	0.506 ± 0.006 (0.499–0.513)	0.093 ± 0.015 (0.074–0.111)
XGBoost	WCM external validation set	0.592 ± 0.008 (0.577–0.608)	0.571 ± 0.009 (0.557–0.584)	0.312 ± 0.007 (0.295–0.329)

On both the CU hold-out test set and WCM external validation set, performance metrics are reported for Random Forest (RF) and XGBoost classifiers developed using three demographic features (age, sex, and race) along with 260 binary ICD-9 code features extracted from the CLINICAL INFORMATION section of CT reports.

AUROC, area under the receiver operating characteristic; CT, computed tomography; CU, Columbia University Irving Medical Center; WCM, Weill Cornell Medical Center.

**Figure 5:**
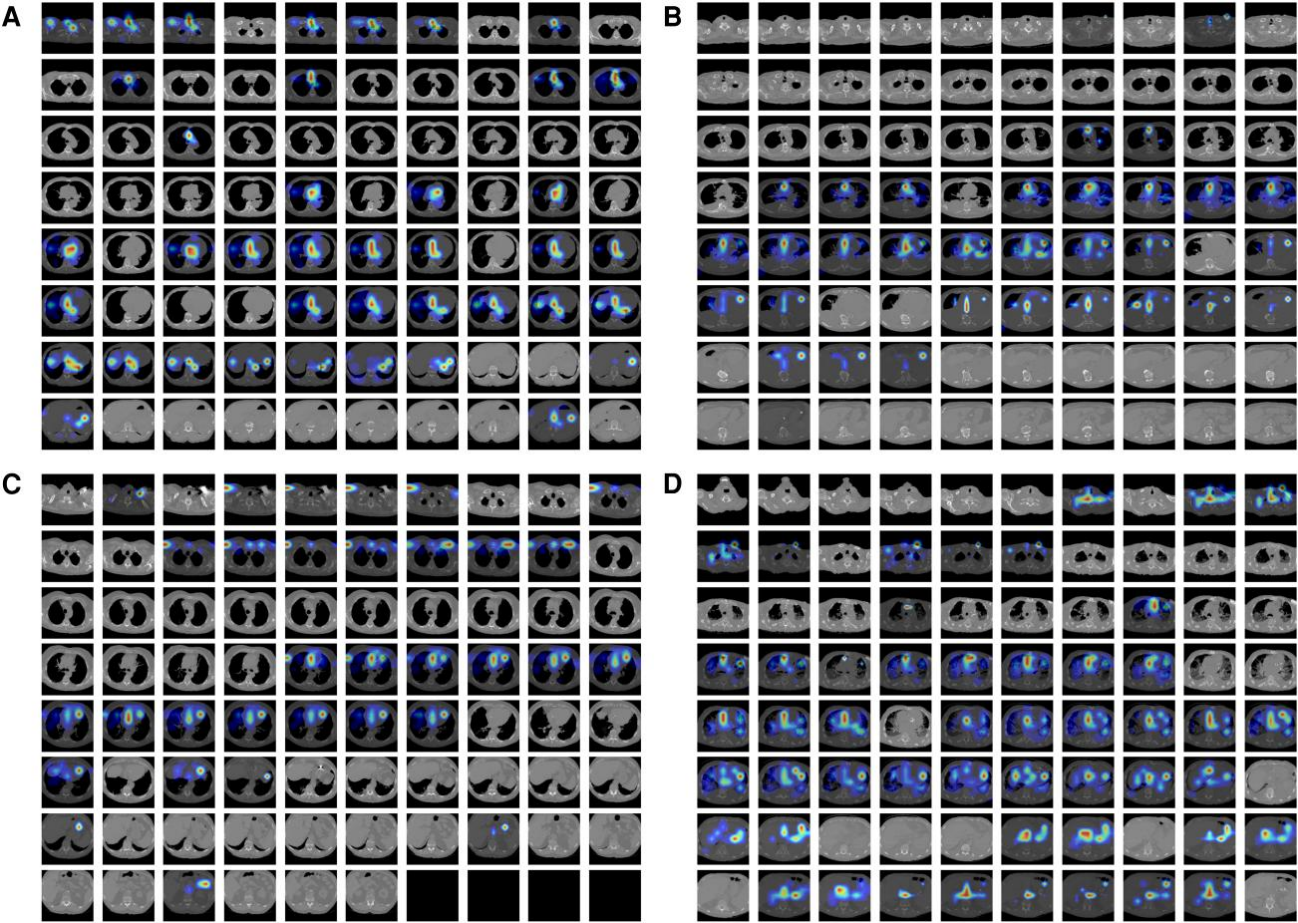
Model interpretability. Sample visualizations of Gradient-weighted Class Activation Mapping saliency maps overlaid on axial-oriented computed tomography scans for four patients with abnormal left-ventricle ejection fraction. These maps highlight regions within the computed tomography scans that contributed most significantly to the artificial intelligence model’s predictions, providing insights into the features used for classification. (*A*) An enlarged heart and dilated superior vena cava reflecting increased venous pressure (due to heart not working hard enough to keep up with venous return. (*B*) Ascending aorta—especially when calcified. (*C*) Presence of pacemakers and pacemaker wires. (*D*) Pulmonary infiltrates from oedema.

In addition to strong discrimination performance, the model demonstrated good calibration. On the CU hold-out test set, the Brier score for the selected model instance was 0.1096, while the average performance across models trained with different random seeds was 0.1120 ± 0.0025 (95% CI: 0.1098–0.1142), indicating consistent calibration performance across training runs. Calibration curves showed close agreement between predicted probabilities and observed outcome frequencies, indicating reliable probability estimation. Calibration curves for all cohorts are shown in *Figure 5*.

### External validation

To evaluate the generalizability of our AI model across different institutions with potentially varied CT data collection protocols, we validated using external data from WCM. This dataset consisted of *n* = 6695 normal study pairs with echo-derived LVEF ≥ 50% and *n* = 1415 abnormal study pairs with echo-derived LVEF <50%. Our AI model achieved an AUROC of 0.762 ± 0.007 (95% CI: 0.753–0.771) and an F1 score of 0.812 ± 0.005 (95% CI: 0.805–0.818). For a range of sensitivity and specificity scores at different thresholding levels on the full ROC curve on the WCM external validation set, see Supplementary material online, *Table S4*.

Calibration performance remained stable on external validation. The model achieved a Brier score of 0.1170 on the WCM dataset, with means ± SD of 0.1199 ± 0.0022 (95% CI: 0.1180–0.1218) across random seeds, supporting generalizability of probability estimates across institutions.

In the demographic-stratified analysis, our AI model exhibited a similar pattern to that observed on the CU hold-out test set. Specifically, our model performed best within the male subgroup with an AUROC of 0.758 ± 0.007 (95% CI: 0.749–0.767), within the senior (65 and above) age subgroup with an AUROC of 0.752 ± 0.008 (95% CI: 0.743–0.762), and within the ANO racial subgroup with an AUROC of 0.781 ± 0.024 (95% CI: 0.751–0.811) (Table c in *Figure 4*). These findings highlight the model’s ability to maintain strong predictive performance across diverse demographic subgroups. Permutation-based one-way ANOVA tests were performed to assess the statistical significance of model performance differences across demographic strata. The performance showed statistical significance between different patient sex subgroups (*P*-value = 0.0256) and across different patient age subgroups (*P*-value = 0.0005). The performance did not show a statistically significant difference across different race subgroups (*P*-value = 0.1162).

In the WCM external validation set, AUROC was higher for the ≤1-day and ≤1-month interval subsets than for the full cohort (≤1 day: 0.782 vs. 0.762, ΔAUROC = 0.020, Holm-adjusted *P* = 0.004; ≤1 month: 0.788 vs. 0.762, ΔAUROC = 0.026, Holm-adjusted *P* = 0.002), whereas the ≤1-week subset was not significantly different from the full cohort (0.769 vs. 0.762; Holm-adjusted *P* = 0.129). Unlike the CU hold-out set, the ≤1-day interval did not show the highest AUROC; the ≤1-month interval had the numerically highest overall AUROC in WCM (*Table 5*).

### Performance of baseline model using demographic and computed tomography report-derived clinical features

We trained both an RF classifier and an XGBoost model using demographic attributes and clinical information (see Methods) on the CU training set as baseline models.

On the CU hold-out test set, the RF model achieved an AUROC of 0.538 ± 0.017 (95% CI: 0.517–0.559) and an F1 score of 0.107 ± 0.018 (95% CI: 0.086–0.131), while the XGBoost model achieved an AUROC of 0.607 ± 0.010 (95% CI: 0.588–0.625) and an F1 score of 0.309 ± 0.009 (95% CI: 0.289–0.330).

On the WCM external validation set, the RF model achieved an AUROC of 0.550 ± 0.013 (95% CI: 0.533–0.565) and an F1 score of 0.093 ± 0.015 (95% CI: 0.074–0.111), whereas the XGBoost model achieved an AUROC of 0.592 ± 0.008 (95% CI: 0.577–0.608) and an F1 score of 0.312 ± 0.007 (95% CI: 0.295–0.329) (*Table 6*).

Both baseline models demonstrated limited predictive performance, with AUROC values only modestly above chance level, highlighting the limited utility of demographic and report-derived clinical features alone in identifying LVEF status. In contrast, the ViT-based AI model leveraging imaging data achieved substantially higher performance.

### Model interpretation

#### Model interpretability for imaging biomarker discovery

**Figure 6:**
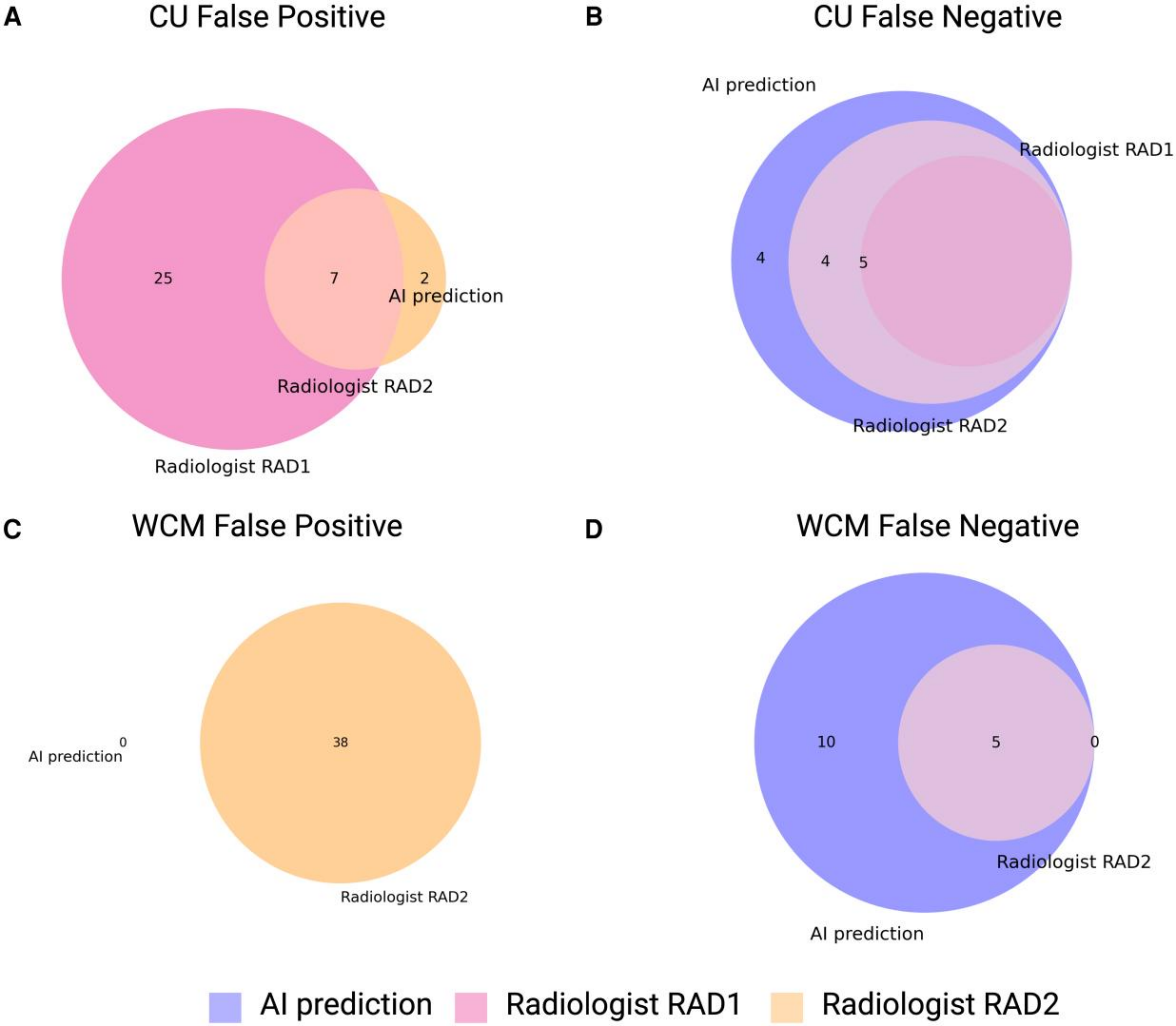
Venn diagrams of error analysis. False positive and false negative results detected by the artificial intelligence model and the two board-certified thoracic radiologists. (*A* and *B*) False positive results and false negative results among the 90 Columbia University Irving Medical Center scans. (*C* and *D*) False positive results and false negative results among the 100 Weill Cornell Medical Center scans.

We applied Grad-CAM^15^ to the CT volume data from both the CU hold-out test set and the WCM external validation set. *Figure 6* displays representative heatmaps overlaid on axial CT slices, illustrating the regions of greatest model activation.

We randomly selected 51 subjects with reduced LVEF (<50%) from the CU hold-out test set for detailed examination by a medical physicist (see Supplementary Data  *S1*). For the reduced EF group, Grad-CAM heatmaps highlighted key cardiopulmonary features associated with low LVEF, including an enlarged heart (e.g. *Figure 6A*), ascending aorta—especially when calcified (e.g. *Figure 6B*), dilated superior vena cava (SVC) reflecting increased venous pressure (e.g. *Figure 6A*), presence of pacemakers and pacemaker wires (e.g. *Figure 6C*), and pulmonary infiltrates from oedema (e.g. *Figure 6D*). Other features include subcutaneous tissue of the back showing increased density from posterior fluid accumulation in HF patients, sternal cerclage wires (indicative of prior cardiac surgery), and neck skin scarring, consistent with previous central line insertion in ICU-treated patients.

#### Comparison with radiologist manual analysis and inference

On the CU scans, the AI model selected based on best validation performance achieved a weighted F1 score of 0.808, compared to 0.646 for RAD1 and 0.802 for RAD2. We note that this comparison is based on a single selected model instance and may therefore reflect modest optimism relative to average model performance across random seeds. On the WCM scans, the AI model’s weighted F1 score was 0.797, surpassing RAD2’s score of 0.623 (*Table 7*). McNemar’s test was conducted to compare the AI model’s predictions with those of two radiologists (RAD1 and RAD2) on the CU hold-out test set, and with RAD2 on the WCM external validation set. Additionally, we compared the predictions of RAD1 and RAD2 on the CU hold-out test set to evaluate inter-radiologist variability. We used the McNemar’s test for paired categorical outcomes, which evaluates whether the proportion of discordant classifications between the two radiologists or one radiologist vs. our AI model differs significantly. Two-sided *P*-values were reported, with statistical significance defined as *P*-value < 0.05. The resulting *P*-values were as follows: between RAD1 and the AI model on CU: *P* = 6.98 × 10^−10^, between RAD2 and the AI model on CU: *P* = 8.74 × 10^−4^, between RAD1 and RAD2 on CU: *P* = 3.02 × 10^−6^, between RAD2 and the AI model on WCM: *P* = 1.17 × 10^−11^. These results demonstrate statistically significant differences in prediction patterns, further emphasizing the distinct prediction capabilities of the AI model relative to individual radiologists, and the significant variability between different radiologists.

**Table 7:** Performance comparison between our AI model and radiologists

	CU *N* = 90 sampled CT scans	WCM *N* = 100 sampled CT scans
RAD1	0.646	—
RAD2	0.802	0.623
AI Model	0.808	0.797

Performance evaluation using F1 score for RAD1, RAD2 and our AI model on randomly selected CT scans from the CU test set and the WCM external validation set.

AI, artificial intelligence; CT, computed tomography; CU, Columbia University Irving Medical Center; WCM, Weill Cornell Medical Center.

This is achieved by our AI model at a faster speed of analysis and inference: approximately 1 min on the 90 CU scans and the 100 WCM scans, respectively, by our AI model vs. ∼2.2 m per CU CT scan by RAD1, and ∼2 m per WCM CT scan by RAD2.

**Figure 7:**
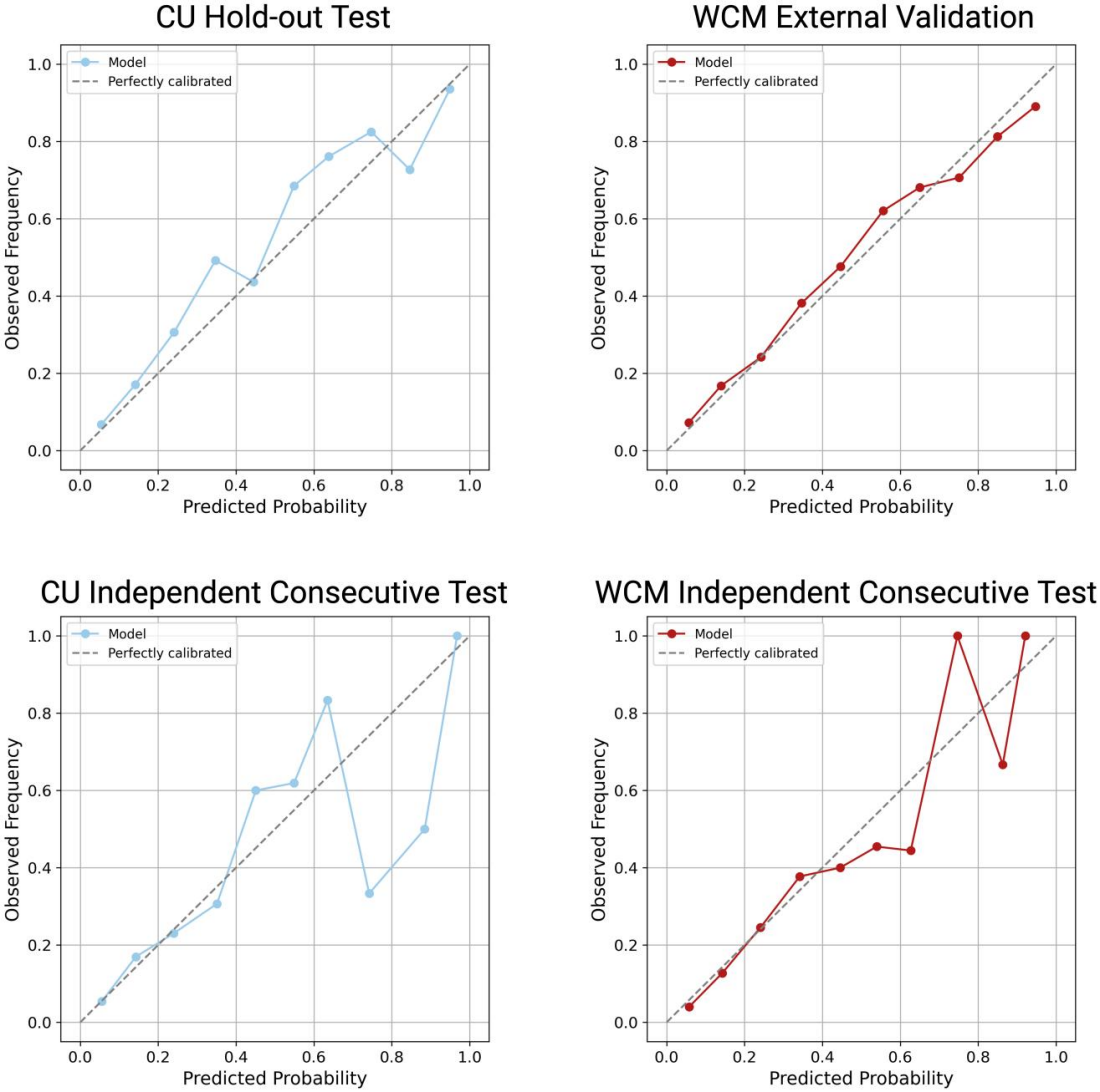
Calibration curves. The computed tomography-to-echo left-ventricle ejection fraction prediction model is evaluated across four cohorts: Columbia University Irving Medical Center hold-out test set, Weill Cornell Medical Center external validation set, and Columbia University Irving Medical Center and Weill Cornell Medical Center independent consecutive test sets. Observed outcome frequencies are plotted against predicted probabilities to assess calibration. The model demonstrates good agreement between predicted and observed risks across cohorts, indicating reliable probability estimation.

Detailed error analysis revealed complementary strengths between the AI model and radiologists. Among the 90 CU scans, the numbers of incorrect predictions were as follows: 13 by the AI model, 37 by RAD1, and 18 by RAD2. Notably, nine errors overlapped between the AI model and one radiologist, while only five errors were shared by the AI model and both radiologists. On the WCM scans, the AI model made 15 incorrect predictions, compared to 43 by RAD2. Errors shared by the AI model and RAD2 totalled five. We summarized the false-positive and false-negative predictions of our AI model and the two radiologists as Venn diagrams in *Figure 7*, offering further insights into the nature of the errors.

##### Evaluation of computed tomography model on the Columbia University Irving Medical Center independent consecutive test set

From the 1895 subjects with 2417 CT studies and 4448 echo reports collected from CU, we curated a CU independent consecutive test set for the LVEF status prediction model comprising 1251 subjects with 1411 pairs of CT–echo studies. Filtering and pairing protocols excluded 484 CT studies and 1006 echo reports to ensure data quality and consistency (see Methods). Notably, the LVEF status distributions stratified by the patient demographics in the CU prospective test set (see Supplementary material online, *Table S8*) were highly consistent with the ones on the primary dataset for our AI model development. The CT series were distributed among three vendors as follows: GE MEDICAL SYSTEMS (1141), Canon Medical Systems (196), and SIEMENS (74). Our AI model trained on the CU primary dataset demonstrated robust performance on the CU independent consecutive test set, achieving an AUROC of 0.742 (95% CI: 0.729–0.755), an F1 score of 0.809 (95% CI: 0.803–0.814), and a balanced accuracy of 0.571 (95% CI: 0.565–0.578) for LVEF status prediction. Performance metrics stratified by age, gender, and race are presented in Supplementary material online, *Figure S1C*. The model maintained good calibration in this prospective cohort, with a Brier score of 0.1102 for the best seed and consistent results 0.1144 ± 0.0029 (95% CI: 0.1119–0.1169) across random seeds.

##### Evaluation of artificial intelligence model on the Weill Cornell Medical Center independent consecutive test set

Using the 1201 subjects with 1366 WCM CT studies and their associated 2377 echo study reports, we curated a WCM prospective test set for the LVEF status prediction model comprising 755 subjects with 817 pairs of CT–echo studies. Based on our filtering and pairing protocols (see Methods), 549 CT studies and 1560 echo study reports were excluded. Notably, the LVEF status distributions stratified by the patient demographics in the WCM independent consecutive test set (see Supplementary material online, *Table S8*) are highly consistent with the ones on the WCM external validation dataset (see Supplementary material online, *Table S8*). The CT series were distributed among two vendors as follows: GE Medical Systems (737) and SIEMENS (80). Our AI model trained on the CU primary dataset demonstrated robust performance on the WCM prospective test set, achieving an AUROC of 0.735 (95% CI: 0.701–0.770), an F1 score of 0.825 (95% CI: 0.818–0.833), and a balanced accuracy of 0.563 (95% CI: 0.546–0.580) for LVEF status prediction. The model performance is detailed in the Supplementary material online, *Figure S1C*, stratified by age, gender, and race. Similarly, calibration performance remained stable in the WCM prospective cohort, with a Brier score of 0.1031 for the best seed and 0.1080 ± 0.0039 (95% CI: 0.1046, 0.1113) across random seeds, indicating reliable risk estimation in a real-world clinical setting.

### Decision-curve analysis

**Figure 8:**
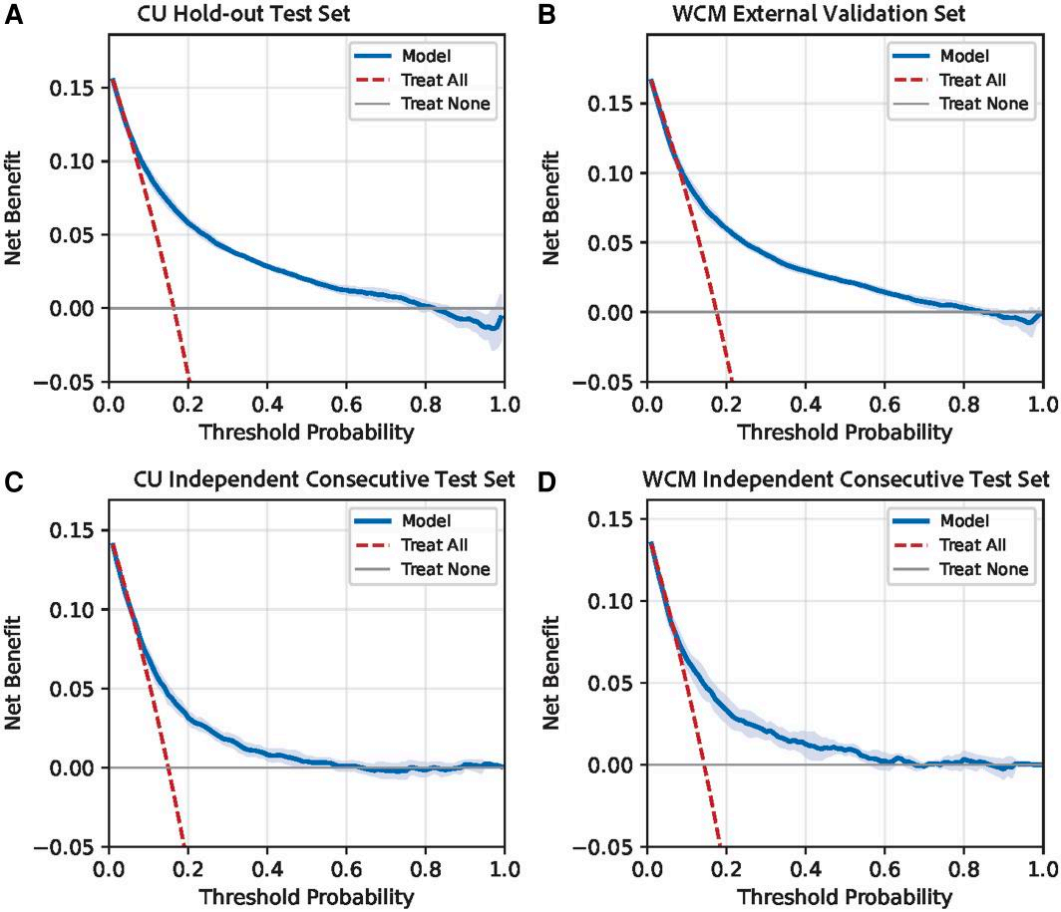
Decision curve analysis. Our computed tomography-to-echo left-ventricle ejection fraction prediction model is compared against ‘Treat All’ and ‘Treat None’ strategies across four cohorts. Net benefit (mean ± 1 std across five seeds) is plotted as a function of threshold probability. The model provides higher net benefit than both baseline strategies primarily within low-to-moderate threshold probabilities, with diminishing benefit at higher thresholds.

Decision-curve analysis showed that the AI model provided higher net benefit than both ‘treat all’ and ‘treat none’ strategies across a range of threshold probabilities in the CU primary test set, WCM external validation test set, CU and WCM independent consecutive test sets (*Figure 8*). The benefit was most pronounced within low-to-moderate threshold probabilities, with diminishing incremental benefit at higher threshold levels. These results suggest that using the AI model to guide follow-up echocardiography could improve clinical decision-making compared with baseline strategies.

### Opportunistic screening

We reviewed another set of 712 chest CT studies of 702 patients, without requiring paired echocardiograms, to assess our model across a broader patient population undergoing routine CT scans. After filtering and preprocessing based on the DICOM metadata and imaging files of the CT data (see Methods), we obtained 633 studies of 627 patients. Our model identified 23 patients (3.7%) for further evaluation with EF < 50%.On further evaluation of clinical documentation of clinical status of these 23 patients identified as abnormal by the model 18/23 (78%) were confirmed to have abnormal EF with a diagnosis of HF. Of the five patients who did not have abnormal EF, two had a previous diagnosis of clinical HF, one had a moderate pericardial effusion, and two had a diagnosis of atrial fibrillation.

## Discussion

In this study, we demonstrated that an AI model can detect abnormal LVEF using non-contrast, non-gated chest CTs ordered for standard clinical indications from two separate sites. The model achieved mean AUROCs of 0.786 at CU and 0.762 at WCM, on retrospective test data. In our independent consecutive test set study with data collected after August 2023, our AI model demonstrated similar performance, achieving a mean AUROC 0.742 of CU and 0.735 of WCM. To our knowledge, this is the first reported study to identify abnormal LVEF, which is traditionally detected from targeted cardiac testing such as echocardiography, multi-gated acquisition scan (MUGA), or cardiac MRI, from standard non-gated non-contrast chest CTs ordered for other medical indications.

The lifetime risk of developing HF is now estimated to be 24% with a prevalence of 1.9% to 2.6%.^2^ Unfortunately, initial gains in HF management in the USA have reversed mortality rates due to HF higher than those in 1999, thought to be driven by increasing incidence of obesity, diabetes mellitus, and hypertension.^16^ About 24–34% of patients are estimated to have Stage B HF (asymptomatic LV dysfunction).^2^ Lifestyle modifications and GDMT have been shown to improve quality of life as well as mortality in this cohort of patients.^17^ There is an unmet need to detect these patients earlier to initiate lifestyle modifications and GDMT. There are currently no clinical screening guidelines for HF recommended in the general population, likely due to the clinical cost of screening using targeted cardiac testing.

Opportunistic screening has been described as the practice of systematically leveraging imaging data that are incidental to the clinical indication of study.^18^ There have been several studies that have attempted to identify abnormal LV structure or function using opportunistic screening. Several studies have used ECG-based AI algorithms to detect LV systolic dysfunction and risk of HF.^19–23^ Bhave *et al.* used a deep learning model to identify patients with severe left ventricular hypertrophy or dilated left ventricle, harbingers of HF, from chest X-rays with a composite AUC of 0.79.^24^ CT provides detailed cross-sectional information and a wealth of biometric data compared to chest X-rays. In a study by Miller *et al.*, a combined AI model using chest CT was able to quantify coronary calcium, left atrial volume, and left ventricular mass index to predict cardiac death or myocardial infarction with an AUC of 0.792.^10^ To our knowledge, our study is the first to utilize AI to determine abnormal LVEF from non-contrast, non-gated chest CT. LVEF has been traditionally determined by cardiac-specific testing in which cardiac dimensions are able to be calculated in both systole and diastole, such as in echocardiography or cardiac MRI, to calculate the percentage of blood ejected with each beat. Calculation of EF using non-gated, non-contrast chest CT presents a novel application of non-gated chest CT that has not been explored previously, and in a previous era potentially not thought to be possible. It is estimated that over 15 million chest CTs are performed in the USA yearly.^25^ Opportunistic screening for abnormal EF may present a unique opportunity in the future for early identification of Stage B HF with a goal to initiate lifestyle measures and initiation of GDMT to prevent progression to Stage C and Stage D HF, HF admissions and mortality.

Deploying this AI-based opportunistic screening tool to identify reduced LVEF introduces important considerations for healthcare delivery. The model, trained on retrospective data, was evaluated across various operating points (see Supplementary material online, *Table S4*). We selected a threshold that prioritized specificity (80% at CU site) in order to minimize false positives and avoid unnecessary downstream testing. At this threshold, sensitivity was 64% (at CU site), enabling identification of the majority of patients with reduced LVEF while reducing the burden of follow-up echocardiography in patients unlikely to have disease. This threshold-dependent pattern is consistent with the intended use of the model as an opportunistic screening tool, where low-to-moderate decision thresholds may be more clinically relevant for identifying patients who warrant confirmatory echocardiographic evaluation. By ensuring a relatively high specificity, we aim to improve the clinical actionability of model outputs and maintain clinician trust while still capturing a meaningful proportion of patients with undiagnosed systolic dysfunction. Using the same threshold, applied across a broader patient population undergoing routine CT scans, without requiring paired echocardiograms, the model identified 23 out of 627 patients for further evaluation. These operating characteristics applied to the prevalence in the test population equate to a number needed to evaluate (NNE) of 3.

While our study demonstrates the diagnostic potential of the CT–LVEF model, several barriers must be addressed to ensure its successful integration into clinical practice. This study does not assess the clinical outcomes of patients found to have an abnormal EF via the model and after subsequent referral for definitive testing confirming an abnormal EF, such as echocardiography. We plan to address this with a prospective study that investigates clinical outcomes in the future. In addition, insurance coverage for follow-up echocardiography may be inconsistent, particularly for asymptomatic patients flagged by an AI algorithm, raising concerns about out-of-pocket costs and payer reimbursement policies. Notably, 3.63% of patients flagged by the model received CT scans for indications that may not justify insurance coverage for follow-up echocardiography, potentially leading to significant out-of-pocket costs. This financial burden is concerning, given that only 37% of US adults can cover an unexpected $400 expense without borrowing, making it vital to consider financial protections for lower-income patients.^26,27^ While echocardiograms are minimal risk to a patient, false positives can lead to unnecessary downstream testing and anxiety, with implications for resource utilization and patient experience. Ethical considerations include ensuring informed consent, protecting patient autonomy in incidental AI findings, and addressing disparities in access to follow-up care. Integrating model outputs into standard CT reporting workflows can increase adoption, particularly among radiologists and clinicians, although optimal implementation may necessitate new operational pathways, including centralized processes to ensure prompt referrals for confirmatory echocardiograms. Finally, training protocols should incorporate these healthcare delivery considerations alongside model information to ensure equitable and effective deployment across diverse populations. These challenges underscore the need for new policy frameworks and clinical trials to better understand the balance of early detection benefits with the risks of overdiagnosis and inequity.

Our AI model was able to extract a signal beyond 2 board certified thoracic radiologists in predicting LVEF status from CT imaging, achieving higher F1 scores on both the CU and WCM test sets. Notably, error analysis revealed that the AI model and radiologists’ mistakes were largely non-overlapping, suggesting that their predictions captured different aspects of the imaging data. Averaging prediction scores from the AI model and radiologists yielded only modest improvements in AUROC (0.729 on CU, 0.719 on WCM) compared to radiologists but fell short compared to pure AI predictions (0.763 on CU, 0.725 on WCM), indicating limited synergy from a simple score combination. Consistent with this, baseline models using demographic and CT report-derived clinical features, including both RF and XGBoost, demonstrated only modest performance (*Table 6*), reinforcing that structured clinical data alone do not capture sufficient signal for accurate LVEF prediction compared with imaging-based approaches.

In this study, we utilized Grad-CAM mapping to evaluate potential imaging biomarkers detected by the AI model to identify abnormal EF. The findings identified through medical physicist review of saliency maps suggest that our model learns biologically and clinically meaningful cues, such as the cardiopulmonary features, which extend beyond core organ structures. These observations highlight the potential of Grad-CAM not only to enhance AI model interpretability but also to uncover previously unrecognized imaging biomarkers that could provide novel insights into HF pathophysiology.

Our study has limitations, for which we outline potential areas for future work. Firstly, the applicability of our AI model is limited due to its retrospective nature. We plan to conduct a prospective study (in contrast to the independent consecutive test set study we conducted) with follow-up sessions to monitor EF status over time. In addition EF calculation by 2D echocardiography has been reported to show intraobserver and interobserver variations of 8–21% and 6–13% respectively.^28^ In this study, LVEF values were derived from routine clinical echocardiography reports based on 2D echocardiographic assessments. The echocardiography data were obtained from clinical imaging systems, syngo (Siemens) and Xcelera (Philips). The number of studies stratified by LVEF operator and echocardiography data source across four test subsets are summarized in *Table 8*. Specific measurement techniques (e.g. Simpson’s biplane method, single-plane method, or visual estimation) were not consistently documented across reports, precluding stratification by calculation method. Consequently, inter-operator variability and methodological heterogeneity may introduce noise into the ground-truth labels. However, this reflects real-world clinical practice and supports the generalizability of our findings to routine care settings. Although we attempted to pair the echo report closely associated with chest CTs in time, variation in EF overtime is also well described. We acknowledge that medication changes or therapeutic interventions during the interval may lead to changes in EF. We plan to extend our study by considering different training data set building strategies when data collected from different real-life scenarios are available (e.g. sufficiently large cohort of patients with same day CT session followed by echo session). Our study only considered non-contrast CT scans to include a broader range of patients and increase dataset size. However, given the distinct features provided by contrast-enhanced CT, such as clearer visualization of blood vessels, we intend to expand our research by training the model on contrast-enhanced CT data. Additionally, our use of Grad-CAM^15^ offers qualitative interpretation in case studies but lacks population-level interpretability, which limits potential insights into novel biomarkers or pathophysiologic mechanisms. We attribute this limitation to the lack of registration between different CT scans for comparing between different CT scans at the voxel-level, and plan to address it by integrating advanced CT registration approaches^29^ to enable spatial alignment across scans and facilitate population-level analysis in our future research. We acknowledge that the current study does not explicitly investigate the impact of including or excluding scans with known artefacts or low quality in training and validations. Since our objective was to develop a model that performs robustly under real-world clinical conditions, including variability in image quality, we made the design choice to not rigorously exclude lower-quality scans during training or validation to facilitate the application of our model to more general CT imaging settings. In the future, we will conduct more rigorous studies to investigate the sensitivity of our model to the known artefacts or low quality of the imaging data. We hope these changes will further improve upon accuracy of the model. Additionally, for certain analyses requiring a single model instance, including radiologist comparison, calibration visualization, and interpretability analyses, we selected the model with the best validation performance among multiple trained models. This approach may introduce a degree of optimism bias compared to reporting average performance across all seeds. Although we mitigated this by reporting mean performance metrics across multiple runs for primary evaluations, future studies should consider fully seed-averaged or ensemble-based approaches for all downstream analyses to further improve robustness.

**Table 8:** Sample counts stratified by LVEF operator and echocardiography data source across four test subsets

Cohort	LVEF Operator	syngo	xcelera	Total
WCM external validation (*N* = 8110)	=	47	5723	5770
	mean	270	1861	2131
	>	6	200	206
	∼	2	0	2
	<	1	0	1
CU primary test (*N* = 5174)	mean	4040	59	4099
	=	788	57	845
	>	115	0	115
	∼	112	0	112
	<	3	0	3
WCM independent consecutive test (*N* = 817)	=	1	500	501
	mean	19	286	305
	>	0	11	11
CU independent consecutive test (*N* = 1411)	mean	981	154	1135
	=	197	36	233
	>	16	10	26
	∼	14	0	14
	<	1	2	3
Data source totals	WCM external validation	326	7784	8110
	CU primary test	5058	116	5174
	WCM independent consecutive test	20	797	817
	CU independent consecutive test	1209	202	1411

Operator values indicate measurement method: ‘=’ for calculated/direct value, ‘mean’ for range measurement (i.e. LVEF = 40–50%), ‘∼’ for approximate value based on systolic function. When an operator of ‘<’ or ‘>’ is used, the final value has 2.5 subtracted from it or added to it, respectively.

CU, Columbia University Irving Medical Center; LVEF, left-ventricle ejection fraction; WCM, Weill Cornell Medical Center.

In conclusion, we developed and validated an AI model that can successfully predict abnormal LVEF status, directly from non-gated non-contrast CT volumetric imaging data. Our study curated a large multi-modality dataset of paired CT–echo studies based on non-contrast CT scans and echo study reports, using data collected from two independent academic medical centres. As the first study of such cross-modality prediction of cardiac disease indicators using chest CT, our model demonstrated promising performance in both the internal validation and the external validation. We also identified possible imaging biomarkers through the study of selected representative CT scans overlaid by saliency maps. This initiative provides empirical and numerical evidence supporting the use of AI-based opportunistic screening in real-world clinical settings to facilitate earlier diagnosis and possible intervention for patients with HfmEF and HFrEF. By analysing chest CT imaging data, this approach may not only optimize diagnostic accuracy for structural cardiac diseases but also lead to the discovery of novel biomarkers in structural features, which could significantly improve patient care and deepen our understanding of cardiac pathophysiology.

### Statistical analysis

The performance of the AI model, with the output being probability scores, were evaluated using the receiver operating characteristic (ROC) curve, and measured with the area under the ROC (AUROC). Sensitivity and specificity pairs tables were evaluated with either one of the two configured with different thresholds and computed the other using the corresponding point on the ROC curve. The binary classification results, both from the AI models and from the radiologist manual reading results, were evaluated using the F1-score (i.e. harmonic mean of Precision and Recall) and balanced accuracy (i.e. the average of sensitivity and specificity) metrics. All metrics were computed through an open-source python library Scikit-learn (sklearn).^30^ The mean results, standard deviations, and confidence intervals were calculated using model weights trained with five different random seeds on the CU training set. To evaluate whether model performance differed across CT–echo time-interval strata, AUROC values for the ≤1-day, ≤1-week, and ≤1-month subsets were compared with the corresponding full cohort AUROC using two-sided Welch *t*-tests based on the reported mean ± standard deviation across five independently trained random seeds. The standard error of the AUROC difference was calculated from the seed-level standard deviations, with degrees of freedom estimated using the Welch-Satterthwaite approximation. *P*-values were adjusted within each cohort for the three interval vs. full-cohort comparisons using the Holm method, with adjusted *P* < 0.05 considered statistically significant. Because the interval-restricted subsets were nested within the full cohort and these comparisons used summary AUROC estimates across random seeds, the interval analyses were considered exploratory. The computations used Python’s native *statistics* library, specifically the *mean* and *stdev* methods. The McNemar’s test for paired categorical outcomes to compare the predictions of RAD1 and RAD2 on the CU hold-out test set to evaluate inter-radiologist variability. Two-sided *P*-values were reported, with statistical significance defined as *P* < 0.05. For analyses requiring a single model instance (e.g. radiologist comparison and visualization), we selected the model with the best validation performance among the five independently trained models. We note that this selection strategy may introduce optimism bias relative to the average performance across seeds; therefore, primary performance metrics are reported as mean ± standard deviation across all seeds to provide a more robust estimate.

## Supplementary Material

ztag088_Supplementary_Data

## Data Availability

The datasets generated and/or analysed during the current study are not publicly available due to institutional policy and human subjects’ approval requirements to protect patient privacy but are available from the corresponding author on reasonable request.

## Code and model weight availability

The code used for data preprocessing, model development, and evaluation, along with the trained model weights, will be made publicly available at GitHub (https://github.com/ZB-WCM/AI_CT_LVEF) upon publication of this manuscript.
