# Supplementary material for: An artificial intelligence model to detect abnormal ejection fraction from non-contrast chest computed tomography: the CT–LVEF study
Source: Eur Heart J Digit Health. 2026 Jun 11;7(6):ztag088. doi: 10.1093/ehjdh/ztag088 (PMC13322390; doi:10.1093/ehjdh/ztag088)
Supplement: ztag088_Supplementary_Data [file ztag088_supplementary_data.docx]

An Artificial Intelligence Model to Detect Abnormal Ejection Fraction from Non-Contrast Chest Computed Tomography: The CT-LVEF study

Supplementary Materials

*Data linkage*

We formed the CT-echo study pairs by linking data from four categories (see Supplementary Files). (1) The CT DICOM file indexing table describes the relationship from each CT study to its series and DICOM filenames, with each filename indexing a single 2D slice of a 3D volume image. (2) The DICOM Linker table matches each CT study with its corresponding order ID. (3) The image report table connects patient demographics and EMPIs with their CT study order IDs. (4) The echo features table includes measurements and observations, particularly LVEF, from echo reports associated with each patient. Specifically, we used the CT study instance UID to link tables (1) and (2), the CT order ID to link tables (2) and (3), and the patient EMPI to link tables (3) and (4). Ultimately, each qualified 3D CT volume is paired with an LVEF value from a corresponding echo study of the same patient.

*Data filtering*

The CT-echo pairs are filtered based on the metadata from both the CT and echo data, and the time interval between the paired CT and echo studies. Specific criteria for CT data, which are applied to the metadata fields extracted from the CT DICOM files, include: the number of slices per series must be at least 10; only images in axial orientation are included, excluding images with ‘secondary’ and ‘localize’ in image description text through string matching; slice thickness must be $\leq$2mm; the examined body part must not be the abdomen; the modality must be CT; the study description should indicate ‘without IV contrast’; and the images with convolution kernels labeled as 'cardiac' are excluded (Supplementary Table 1). For the echo data, only transthoracic echo (TTE) studies with non-empty LVEF values are included in the report. Any studies from patients with left ventricular assist devices (LVAD) or right ventricular assist devices (RVAD) were excluded. Additionally, patients with multiple echo studies conducted within a 72-hour period were also excluded. This ensures relevance and consistency in the paired data.

*3D non-contrast chest CT volume preprocessing: focusing on the patient’s body region*

The following steps of masking were conducted to focus on the patient’s body region: (i) Convert RGB to grayscale if necessary. (ii) Use OTSU thresholding to generate a binary mask. (iii) Identify the largest connected component as the patient's body region. (iv) Remove small objects and fill holes to clean the mask. (v) Apply this refined mask to remove the bed and other background elements, focusing the crop on the patient’s body region.

*Details of our AI model: Classifier based on a Pretrained CT-ViT Encoder*

We used a vision transformer architecture^1^: the encoder of the CT-ViT framework from GenerateCT^2^, as our backbone model for feature learning from the 3D CT images. We utilized the pre-trained weights of this encoder^2^ and further trained it on our training cohort where both CT and echo studies were from CU. This encoder takes a preprocessed 3D CT volume image of size 164 x 164 x 164 and randomly cropped into $164\times144\times144$ (dimensions are depth, height and width: $Z\times H\times W$) as input and output a $512$-dimensional feature vector. The CT-ViT encoder consists of three modules: the patch embedding layer, the spatial transformer module and the causal transformer module. The patch embedding layer first extracts non-overlapping patches of $2\times16\times16$ from the 3D CT volume input. Each patch is then transformed into a $512$-dimensional feature vector (dimension in tensor: $D$) with a fully connected layer. This transformation yields a $(\frac{164}{2}) \times(\frac{144}{16})\times(\frac{144}{16}) \times512$ feature tensor for each input 3D image. This tensor is then fed into the spatial and temporal transformer. Subsequently in the spatial transformer module, multiple transformer layers were applied with self-attention along the spatial dimensions (i.e., W and H) of the reshaped tensor of $(\frac{164}{2}) \times(\frac{144}{16}\times\frac{144}{16}) \times512$ (dimensions: $Z\times(H\times W)\times D$). This is followed by the causal transformer module where multiple transformer layers were applied over the temporal (i.e., Z) dimension of the reshaped tensor of$(\frac{144}{16}\times\frac{144}{16}) \times(\frac{164}{2}) \times512$ (dimensions: $(H\times W)\times Z\times D$) with causal self-attention such that each spatial token only observes spatial tokens from previous slices in an auto-regressive manner. The dimensionality of output is retained after each spatial and causal transformer layer, ensuring that the volumetric information is preserved throughout the model fine-tuning. Finally, an average pooling over spatial (W and H) and temporal (Z) dimension of output tensor was applied to obtain the output $512$-dimensional feature vector for each input. A fully connected layer is used as a classification head and outputs a univariate prediction as a probability for the input 3D image to be a positive sample.

For predicting binarized LVEF, the AI model were trained to minimize the Binary Cross Entropy loss between the prediction and the binarized LVEF derived from echo report in Eq. 1, where the i-th input preprocessed 3D CT volume is denoted $x_{i}$, its binarized LVEF derived from echo report $y_{i}$, and the prediction made by our framework based on CT-ViT encoder $\hat{y}_{i}$, the total number of training instances $N$.

$L_{BCE}(x_{i},y_{i}, \hat{y}_{i}) = -\sum_{i=1}^{N} y_{i}log{(\hat{y}}_{i}) + (1 - y_{i})log(1-\hat{y}_{i})$ (1)

We used the AdamW optimizer with an initial learning rate of $10^{-5}$, weight decay of $10^{-4}$ and batch size of 8 for 20 epochs. The learning rate decayed to half at epoch 15. During training, random cropping from $164\times164\times164$ to $164\times144\times144$ and random horizontal flipping were used for data augmentation. During testing, center cropping from $164\times164\times164$ to $164\times144\times144$ were used. To assess the model's behavior and reliability, we trained it using the CU training and validation sets five times using different random seeds, resulting in five distinct sets of model weights. The AI model was trained and evaluated with a single NVIDIA A100 GPU.

### *Processing Time*

We evaluated runtime efficiency on virtual CPU and GPU resources provided by Microsoft Azure using 50 samples. Experiments were conducted on virtual CPUs (AMD EPYC 7V12, x86-64) and virtual GPUs (Tesla V100). On the CPU, the average preprocessing time per sample was approximately 40 seconds, with a total inference time—including data loading, processing, and model execution—of around 16.5 minutes. On the GPU, preprocessing time per sample was reduced to approximately 25 seconds, and total inference time was significantly faster at approximately 3.3 minutes.

**
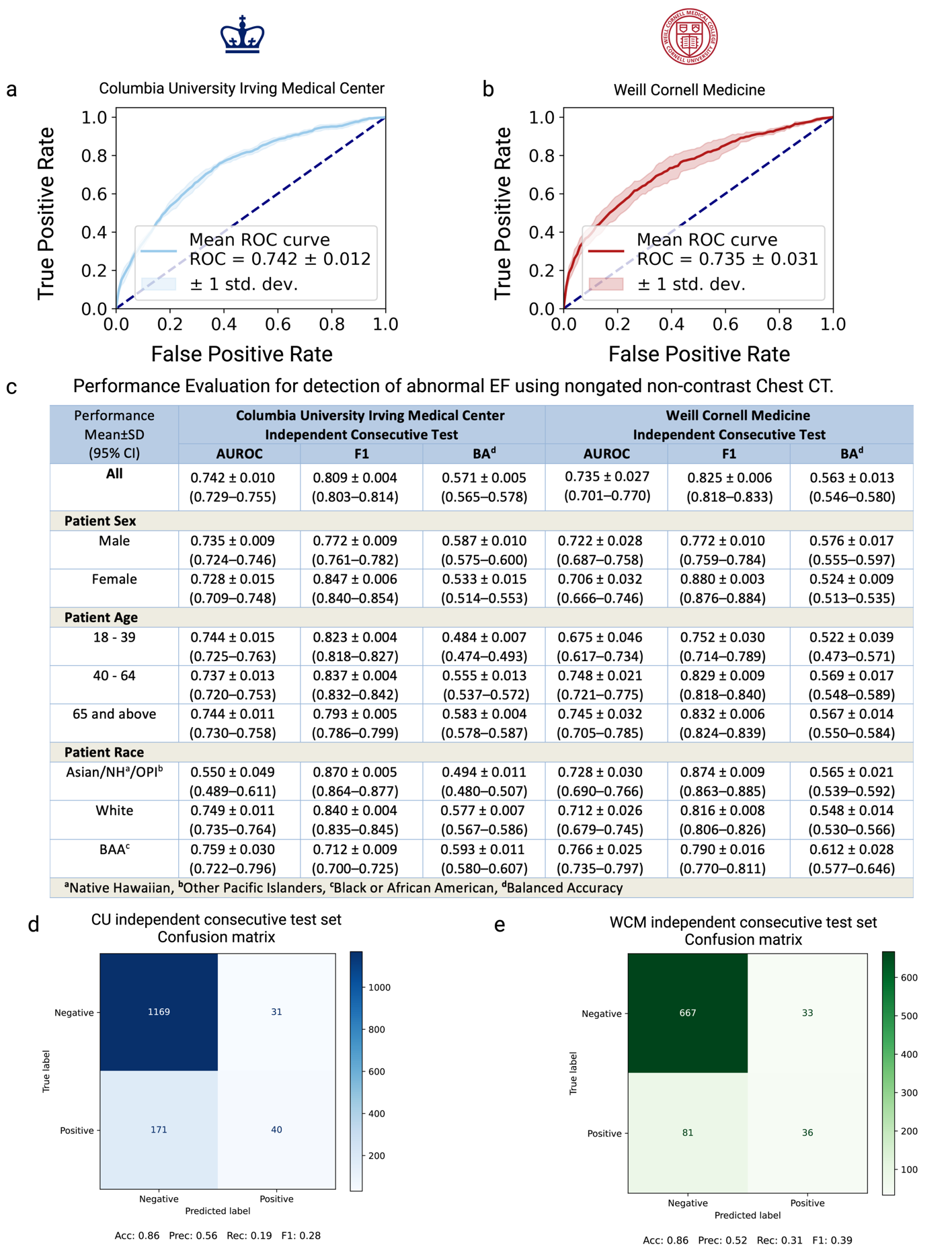
**

**Supplementary Figure 1. Performance Evaluation on independent consecutive test sets: Detection of abnormal EF using non-gated non-contrast Chest CT. a and b. Site-specific test set performance evaluation using ROC and AUROC for CU independent consecutive test set (a) and WCM independent consecutive test set (b) respectively. c. Performance evaluation in AUROC, F1-score and Balanced Accuracy for subpopulations stratified from the test set according to site and demographic attributes: gender, age and race. See Supplementary Table 4 for corresponding population statistics of independent consecutive test set study cohorts.**

**
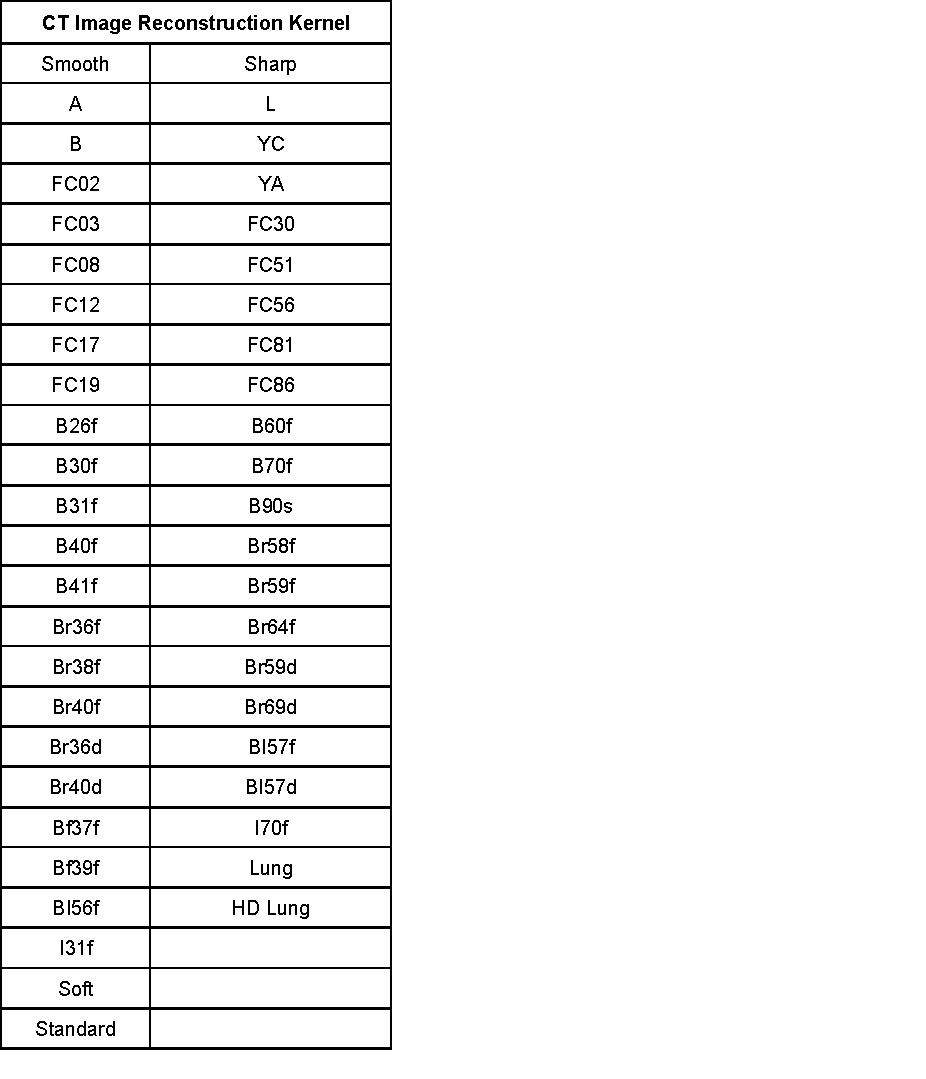
**

**Supplementary Table 1: Convolution Kernel**

## **Definition**

### **Syngo**

For Syngo, the following priority waterfall logic was used:

- Single % values from findings (cumc_ejection_fraction_obs or ejection_fraction est_obs)
- % range from findings
- Calculated measurement value from measurements (lv_ef_mod_bp_calc)
- If no calculated measurement is made, the follow approximations will be made based on the systolic function finding (systolic_function_obs or cumc_systolic_function_edited_obs). The operator will be set to '~' to indicate an aproximation/imputation



 'hyperdynamic' = 72.5



 ['normal','global_normal'] = 60



 'borderline' = 52.5



 'mild' = 47.5



 ['mild-moderate','mild-to-moderate','mildly_decreased'] = 42.5



 ['moderate','moderately_decr'] = 37.5



 ['moderate-severe','mod_to_sev_decr'] = 30



 ['severe','severe_decrease'] = 22.5

### **Xcelera**

For Xcelera the following priority waterfall logioc was used:

- Single, numeric values recorded in under numerous finding names
- Any remaining numeric values that appear as a range in



 Bad ranges and/or parsings will be excluded

- If there are multiple measurements captured through findings at this point, the maximum value will be retained.
- Any categorical assessments of LVEF in
- Any LVEF measurements of menthod mod-bp or Teich



 bp-mod measurements will be prioritized over Teich

NOTE: There are none as of 2/28/2023



 Measurements with the greatest frequency will be prioritized it there are multiple within that type in a given study



 Only measurements between 0 and 100 included

Xcelera

s that the feature

s were generated from:

- 'stress ef range _','ef simpson''s fill _','normal lv size, wm ef est 60-65%','normal lv size, wm ef est _%', 'moderate to severely abnl lv ef estimated 30-35%','mild to moderately abnl lv ef estimated at 40-45%','mildly abnormal lv ef estimated at 45-50%', 'moderately abnl lv ef estimated at 35-40%','borderline normal lv ef estimated at 50-55%','cannot assess ef','severely abnl lv ef estimated at _%', 'lv normal _ %','automated lvef =

_%','lv ef _% by 3d volume','visually est ef = _%.','hyperdynamic lv ef estimated greater than 75%','ef = _.', '3d ef _.','lvef using the simpson’s biplane method is _','ef visual _','ef range _','ef est _%','calculated ejection fraction is _.','nl wm and syst fn, ef est _%', 'grossly normal ef','ef simpson''s _','normal lv ef estimated at

_%','est ef = _%.','normal ef','ef _%','est ef = _(choose abnormal)%.','ef est _', 'est ef = _(choose normal)%.','ef

= _%.','est ef = _(free text)%.','calculated ejection fraction is _%.'

For any value ranges, the mean value is used (ie 50-55% becomes 52.5%).

When an operator of < or > are used, the final value has 2.5 subtracted from it or added to it, respectively.

|  |  |
| --- | --- |
|  |  |

## **Sources Used**

Tables:

- cradle.echo_findings
- cradle.echo_measurements Output:
- cradle.echo_features_lvef



 study_key



 lvef_value



 lvef_range_min



 lvef_range_max



 lvef_operator



 lvef_source_type



 lvef_source_value

|  | 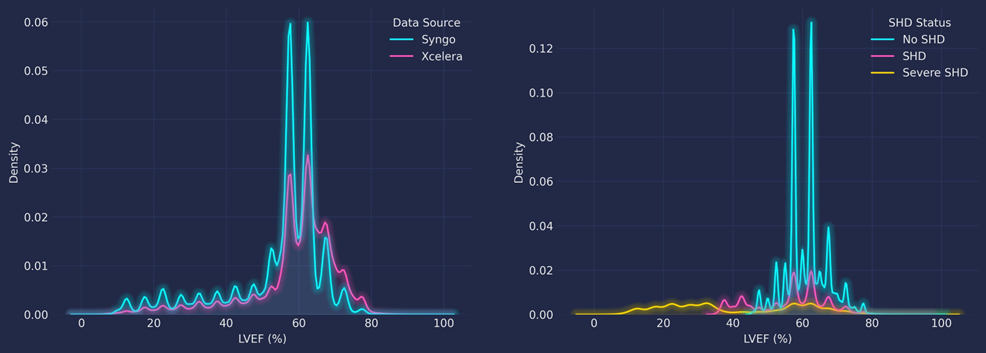 |
| --- | --- |
|  |  |

|  | **lvef_value** |
| --- | --- |
| count | 1.07412e+06 |
| mean | 56.3112 |
| std | 13.1486 |
| min | 0 |
| 25% | 55 |
| 50% | 59 |
| 75% | 62.5 |
| max | 100 |
| percent_missing | 0.418375 |

**Supplementary Table 2: Stepwise algorithm for Determination of Ejection Fraction from Echocardiography Reports**

| **Characteristic** | **CU Train** | **CU Val** | **CU Test** | **WCM External** |
| --- | --- | --- | --- | --- |
| **N** | 19948 | 2805 | 5703 | 8483 |
| **Age, years (mean ± SD)** | 64.0 ± 15.1 | 63.3 ± 15.3 | 63.5 ± 15.7 | 67.6 ± 14.6 |
| **Age, years (median [IQR])** | 66.0 [56.0, 74.0] | 65.0 [55.0, 74.0] | 66.0 [55.0, 74.0] | 69.0 [59.0, 78.0] |
| **Sex** |  |  |  |  |
| **Male, n (%)** | 10173 (51.0%) | 1447 (51.6%) | 2835 (49.7%) | 4365 (51.5%) |
| **Female, n (%)** | 8324 (41.7%) | 1189 (42.4%) | 2485 (43.6%) | 4115 (48.5%) |
| **Other/Unknown, n (%)** | 1451 (7.3%) | 169 (6.0%) | 383 (6.7%) | 3 (0.0%) |
| **Race** |  |  |  |  |
| **White, n (%)** | 9605 (48.2%) | 1278 (45.6%) | 2837 (49.7%) | 4500 (53.0%) |
| **Other combinations not described, n (%)** | 3013 (15.1%) | 517 (18.4%) | 919 (16.1%) | 1043 (12.3%) |
| **Black or african american, n (%)** | 2899 (14.5%) | 435 (15.5%) | 816 (14.3%) | 1162 (13.7%) |
| **Declined, n (%)** | 1990 (10.0%) | 204 (7.3%) | 488 (8.6%) | 1232 (14.5%) |
| **Asian, n (%)** | 676 (3.4%) | 83 (3.0%) | 178 (3.1%) | 506 (6.0%) |
| **Nat hawaiian / pacific island, n (%)** | 80 (0.4%) | 4 (0.1%) | 37 (0.6%) | 8 (0.1%) |
| **Missing/Unknown, n (%)** | 1640 (8.2%) | 279 (9.9%) | 417 (7.3%) | 18 (0.2%) |
| **LVEF < 50%, n (%)** | 3143 (15.8%) | 462 (16.5%) | 931 (16.3%) | 1467 (17.3%) |
| **LVEF, % (mean ± SD)** | 55.7 ± 11.2 | 55.3 ± 11.8 | 55.6 ± 11.2 | 58.7 ± 12.6 |
| **Acquisition Parameter** |  |  |  |  |
| **X-Ray Tube Current, mean ± SD** | 258.2 ± 165.5 | 254.1 ± 162.2 | 253.3 ± 164.1 | 274.7 ± 131.1 |
| **Slice Thickness, mean ± SD** | 1.16 ± 0.27 | 1.15 ± 0.27 | 1.15 ± 0.26 | 1.01 ± 0.30 |
| **KVp, mean ± SD** | 119.8 ± 3.5 | 119.8 ± 3.6 | 119.8 ± 3.4 | 103.2 ± 8.2 |

**Supplementary Table 3: Baseline demographic and imaging characteristics across the CU training, validation, test, and WCM external cohorts.**

| **Sensitivity Threshold** | | | | |
| --- | --- | --- | --- | --- |
| **Sensitivity** | **Specificity_Columbia** | **Specificity_WCM** | **PPV_Columbia** | **PPV_WCM** |
| **0.1** | **0.99** | **0.99** | **0.83** | **0.83** |
| **0.15** | **0.99** | **0.99** | **0.79** | **0.79** |
| **0.2** | **0.98** | **0.98** | **0.67** | **0.7** |
| **0.25** | **0.97** | **0.97** | **0.643** | **0.66** |
| **0.3** | **0.96** | **0.96** | **0.59** | **0.59** |
| **0.35** | **0.94** | **0.94** | **0.53** | **0.51** |
| **0.4** | **0.93** | **0.91** | **0.51** | **0.47** |
| **0.45** | **0.91** | **0.89** | **0.48** | **0.44** |
| **0.5** | **0.88** | **0.85** | **0.44** | **0.39** |
| **0.55** | **0.84** | **0.82** | **0.4** | **0.36** |
| **0.6** | **0.8** | **0.77** | **0.37** | **0.33** |
| **0.65** | **0.76** | **0.72** | **0.34** | **0.3** |
| **0.7** | **0.71** | **0.65** | **0.32** | **0.28** |
| **0.75** | **0.65** | **0.6** | **0.29** | **0.26** |
| **0.8** | **0.58** | **0.53** | **0.27** | **0.25** |
| **0.85** | **0.49** | **0.44** | **0.24** | **0.23** |
| **0.9** | **0.38** | **0.36** | **0.22** | **0.21** |
| **0.95** | **0.27** | **0.26** | **0.19** | **0.19** |

| **Sensitivity Threshold** | | | | |
| --- | --- | --- | --- | --- |
| **Sensitivity** | **Specificity_Columbia** | **PPV_Columbia** | **Specificity_WCM** | **PPV_WCM** |
| **0.1** | **0.996018** | **0.827503** | **0.996172** | **0.833092** |
| **0.15** | **0.992245** | **0.787014** | **0.992445** | **0.791359** |
| **0.2** | **0.981555** | **0.674428** | **0.983464** | **0.697943** |
| **0.25** | **0.97359** | **0.643934** | **0.974857** | **0.655125** |
| **0.3** | **0.959966** | **0.588752** | **0.961264** | **0.596706** |
| **0.35** | **0.942779** | **0.538862** | **0.936247** | **0.511915** |
| **0.4** | **0.927269** | **0.512358** | **0.914127** | **0.470868** |
| **0.45** | **0.906414** | **0.47879** | **0.892188** | **0.443642** |
| **0.5** | **0.878223** | **0.439586** | **0.853956** | **0.395427** |
| **0.55** | **0.844267** | **0.402881** | **0.816536** | **0.36416** |
| **0.6** | **0.804234** | **0.369294** | **0.772402** | **0.334944** |
| **0.65** | **0.757598** | **0.338748** | **0.716892** | **0.304892** |
| **0.7** | **0.708656** | **0.314605** | **0.654754** | **0.2792** |
| **0.75** | **0.645148** | **0.287638** | **0.600107** | **0.263787** |
| **0.8** | **0.577447** | **0.26562** | **0.529779** | **0.245299** |
| **0.85** | **0.485538** | **0.239916** | **0.442673** | **0.225627** |
| **0.9** | **0.380843** | **0.217343** | **0.355538** | **0.210606** |
| **0.95** | **0.267449** | **0.198559** | **0.261397** | **0.197253** |

| **Specificity Threshold** | | | | |
| --- | --- | --- | --- | --- |
| **Specificity** | **Sensitivity_Columbia** | **Sensitivity_WCM** | **PPV_Columbia** | **PPV_WCM** |
| **0.1** | **0.98** | **0.99** | **0.17** | **0.17** |
| **0.15** | **0.97** | **0.98** | **0.18** | **0.18** |
| **0.2** | **0.96** | **0.96** | **0.19** | **0.19** |
| **0.25** | **0.96** | **0.95** | **0.19** | **0.19** |
| **0.3** | **0.94** | **0.93** | **0.2** | **0.2** |
| **0.35** | **0.92** | **0.9** | **0.21** | **0.21** |
| **0.4** | **0.89** | **0.87** | **0.22** | **0.22** |
| **0.45** | **0.86** | **0.85** | **0.23** | **0.23** |
| **0.5** | **0.84** | **0.82** | **0.24** | **0.24** |
| **0.55** | **0.82** | **0.78** | **0.26** | **0.25** |
| **0.6** | **0.79** | **0.75** | **0.27** | **0.26** |
| **0.65** | **0.75** | **0.7** | **0.29** | **0.28** |
| **0.7** | **0.7** | **0.66** | **0.31** | **0.29** |
| **0.75** | **0.66** | **0.62** | **0.34** | **0.32** |
| **0.8** | **0.61** | **0.57** | **0.37** | **0.35** |
| **0.85** | **0.55** | **0.51** | **0.41** | **0.39** |
| **0.9** | **0.46** | **0.44** | **0.47** | **0.45** |
| **0.95** | **0.33** | **0.32** | **0.56** | **0.55** |

| **Specificity Threshold** | | | | |
| --- | --- | --- | --- | --- |
| **Specificity** | **Sensitivity_Columbia** | **PPV_Columbia** | **Sensitivity_WCM** | **PPV_WCM** |
| **0.1** | **0.976359** | **0.171673** | **0.980919** | **0.172336** |
| **0.15** | **0.964539** | **0.178163** | **0.972438** | **0.179361** |
| **0.2** | **0.95918** | **0.186368** | **0.958304** | **0.186229** |
| **0.25** | **0.950355** | **0.194898** | **0.939735** | **0.193141** |
| **0.3** | **0.943262** | **0.20473** | **0.923675** | **0.201334** |
| **0.35** | **0.923168** | **0.213423** | **0.90742** | **0.210549** |
| **0.4** | **0.907801** | **0.224234** | **0.881272** | **0.219117** |
| **0.45** | **0.890071** | **0.236156** | **0.856537** | **0.229299** |
| **0.5** | **0.862096** | **0.247778** | **0.829505** | **0.240666** |
| **0.55** | **0.84279** | **0.263514** | **0.800707** | **0.253694** |
| **0.6** | **0.808511** | **0.278578** | **0.772438** | **0.269499** |
| **0.65** | **0.78487** | **0.299922** | **0.739929** | **0.287689** |
| **0.7** | **0.736028** | **0.319131** | **0.708834** | **0.311007** |
| **0.75** | **0.687943** | **0.344567** | **0.664841** | **0.336894** |
| **0.8** | **0.63948** | **0.379207** | **0.60318** | **0.36555** |
| **0.85** | **0.579196** | **0.424519** | **0.522968** | **0.399783** |
| **0.9** | **0.502364** | **0.489726** | **0.451237** | **0.46296** |
| **0.95** | **0.395981** | **0.602068** | **0.341343** | **0.566015** |

**Supplementary Table 4: Retrospective sensitivity and specificity thresholds**

| Evaluation Metrics | Columbia Test Set | | | | | | |
| --- | --- | --- | --- | --- | --- | --- | --- |
|  | 3 Class Classification | | | 4 Class Classification | | | |
|  | 0 - 39 % | 40 - 69 % | > 70% | 0 - 39 % | 40 - 49 % | 49 - 69 % | > 70 % |
| AUC | 0.453 | | | 0.244 | | | |
| F1 Score | 0.092 | | | 0.021 | | | |
| Balanced Accuracy | 0.479 | | | 0.424 | | | |

| Evaluation Metrics | Cornell Test Set | | | | | | |
| --- | --- | --- | --- | --- | --- | --- | --- |
|  | 3 Class Classification | | | 4 Class Classification | | | |
|  | 0 - 39 % | 40 - 69 % | > 70% | 0 - 39 % | 40 - 49 % | 49 - 69 % | > 70 % |
| AUC | 0.412 | | | 0.371 | | | |
| F1 Score | 0.021 | | | 0.036 | | | |
| Balanced Accuracy | 0.477 | | | 0.447 | | | |

**Supplementary Table 5: Evaluation of Test set performance using different LVEF thresholding schemes for multi-class classification.**

| Evaluation Metrics | Columbia Test Set | |
| --- | --- | --- |
|  | < 40 % | < 35 % |
| AUC | 0.823 | 0.846 |
| F1 Score | 0.372 | 0.396 |
| Balanced Accuracy | 0.621 | 0.642 |

| Evaluation Metrics | Cornell Test Set | |
| --- | --- | --- |
|  | < 40 % | < 35 % |
| AUC | 0.824 | 0.85 |
| F1 Score | 0.389 | 0.4 |
| Balanced Accuracy | 0.641 | 0.664 |

**Supplementary Table 6: Evaluation of Test set performance on different LVEF cut-offs.**

| **Cohort** | **Group** | **N** | **N_pos** | **AUC (mean ± std)** |
| --- | --- | --- | --- | --- |
| **CU Hold-out Test** | CT first | 2,424 | 351 | 0.775 ± 0.007 |
|  | Echo first | 2,750 | 495 | 0.793 ± 0.004 |
| **WCM External Validation** | CT first | 4,134 | 753 | 0.737 ± 0.007 |
|  | Echo first | 3,976 | 662 | 0.786 ± 0.005 |
| **CU Independent Consecutive Test** | CT first | 600 | 90 | 0.709 ± 0.013 |
|  | Echo first | 811 | 121 | 0.749 ± 0.018 |
| **WCM Independent Consecutive Test** | CT first | 384 | 62 | 0.719 ± 0.032 |
|  | Echo first | 433 | 55 | 0.749 ± 0.022 |

**Supplementary Table 7: AUC (mean ± standard deviation across 5 seeds) for LVEF prediction stratified by temporal ordering of imaging, defined as whether the CT was performed before the echocardiogram (CT first) or after (Echo first).**

|  | **Columbia University Irving Medical Center (CU)**  **Total: 1,411 chest CTs** | | **Weill Cornell Medical College**  **(WCM)**  **Total: 817 chest CTs** | |
| --- | --- | --- | --- | --- |
|  | **Normal (LVEF≥50)**  **1,200 (85.04%)** | **Abnormal (LVEF<50)**  **211 (14.95%)** | **Normal (LVEF≥50)**  **700 (85.67%)** | **Abnormal (LVEF<50)**  **117 (14.32%)** |
| **Patient Sex*** | | | | |
| Male | 590(479.16%) | 133 (63.03%) | 333(47.57%) | 82(70.09%) |
| Female | 610(50.83%) | 78(36.96%) | 367 (52.43%) | 35 (29.91%) |
| **Patient Age(years)** | | | | |
| 18 - 39 | 93 (7.75%) | 13 (6.16%) | 27(3.85%) | 06 (5.13%) |
| 40 - 64 | 377 (31.42%) | 53 (25.11%) | 190 (27.14%) | 33 (28.21%) |
| 65 and above | 730 (60.83%) | 145 (68.72%) | 480(68.57%) | 78 (66.67%) |
| **Patient Race*** | | | | |
| Asian/NH/OPI | 33 (2.75%) | 03 (1.42%) | 87 (12.43%) | 10 (8.55%) |
| White | 600 (50.0%) | 84 (39.81%) | 362 (51.71%) | 61 (52.14%) |
| BAA | 204 (17.0%) | 66 (31.27%) | 89 (12.71%) | 23 (119.66%) |

**Supplementary Table 8: Patient Demographics for studies on the independent consecutive test sets.**

**References**

1. Dosovitskiy, A. *et al.* An Image is Worth 16x16 Words: Transformers for Image Recognition at Scale. (2020).

2. Hamamci, I. E. *et al.* GenerateCT: Text-Conditional Generation of 3D Chest CT Volumes. (2023).

## Extended Reporting Checklist for Researchers

| **Section** | **Checklist item** | **Page #** |
| --- | --- | --- |
| **1** | **Designing an AI Study in Cardiovascular Imaging** |  |
| *1.1* | *Appropriateness of applying AI* |  |
| a | Describe the need for applying AI  AI is used to enable opportunistic screening of abnormal LVEF from routinely acquired non-gated chest CT scans, addressing underdiagnosis of asymptomatic systolic dysfunction. | Introduction (p. 4) |
| b | Determine the appropriateness of applying AI  AI is appropriate given the high-dimensional imaging data and the absence of human-interpretable features for EF estimation from non-gated CT. | Introduction (p. 4) |
| *1.2* | *Study objectives, input data type, and prediction target* |  |
| a | Explain the AI task and the likely deployment context  Binary classification of abnormal LVEF (EF<50%) from chest CT for opportunistic screening. | Introduction (p. 4) |
| b | Describe the input data, number of training/test examples  3D non-contrast chest CT volumes; 34,058 CT–echo pairs across two institutions. | Methods – Data Curation and Preprocessing (p. 5) |
| c | Specify model supervision type  Supervised learning using echo-derived LVEF labels. | Methods – Evaluation metrics (pp. 6-7) |
| d | Describe the nature of the model’s output and what it represents  Predicted probability of abnormal LVEF (EF<50%). | Methods – AI model (pp. 5-6) |
| *1.3* | *Design of the AI study* |  |
| a | Describe the study design  Retrospective multi-center study with external validation, consecutive independent set validation, opportunistic screening test. | Methods – Data Curation and Preprocessing (p. 5) |
| b | Describe data origin  CU (training/validation/test, consecutive independent test and opportunistic screening), WCM (external validation and consecutive independent test). | Methods – Data Curation and Preprocessing (p. 5) |
| c | Describe if impact analysis was included  Decision-curve analysis performed to evaluate clinical utility. | Methods – Decision Curve Analysis (p. 8), Results – Decision-Curve Analysis (p. 13) |
| **2** | **Data Format and Preprocessing** |  |
| *2.1* | *Data format* |  |
| a | Describe the technical details of the data acquisition  Non-gated, non-contrast chest CT (DICOM), multi-vendor, multi-center. | Methods – Data Curation and Preprocessing (p. 5) |
| b | Describe the technical details of the data format  3D volumes in Hounsfield Units, resampled to standardized resolution. | Methods – CT preprocessing (p. 5) |
| *2.2* | *Clinical characteristics of the study cohort* |  |
| a | Present the age, sex, and race/ethnicity distributions of the cohort(s)  Age, sex, race distributions reported (Table 1). | Results – Table 1 (pp. 27-28) |
| b | Summarize key clinical, treatment, and imaging characteristics of the cohort(s)  CT acquisition parameters (kVp, tube current, slice thickness) reported (Table 2). | Results – Table 2 (pp. 28-29) |
| c | Compare summary statistics of cases and controls  EF<50% prevalence and subgroup comparisons provided. | Results – Table 1 (pp. 27-28) |
| *2.3* | *Steps of data preprocessing* |  |
| a | Describe how data were cleaned, made uniform, and consistent  Filtering, CT–echo pairing, HU normalization, body masking. | Methods – Data Curation and Preprocessing (p. 5) |
| b | Describe data harmonization techniques (if applicable)  Voxel resampling and intensity windowing applied. | Methods – CT preprocessing (p. 5) |
| c | Provide details on missing values and imputation methods  Handled via dataset curation. No imputation. | Methods – Data Curation and Preprocessing (p. 5) |
| d | Describe processes for handling outliers  Implicitly addressed via preprocessing and inclusion criteria. | Methods – Data Curation and Preprocessing (p. 5) |
| e | Describe whether class imbalance exists  Present (~16–17% abnormal EF), addressed via loss function and evaluation metrics. | Results – Table 1 (pp. 27-28), Results text (p. 8) |
| *2.4* | *Feature engineering and feature selection* |  |
| a | Describe applied feature engineering techniques  3D CT volumes processed via ViT encoder. | Methods – AI model (pp. 5-6) |
| b | Describe applied feature selection techniques  Learned automatically via deep learning (no manual selection). | Methods – AI model (pp. 5-6) |
| **3** | **Selection of AI Methods and Applications** |  |
| *3.1* | *Selecting appropriate AI methods and applications: Clearly define data composition (structured/unstructured)*  Unstructured imaging data (3D CT volumes). | Methods – AI model (pp. 5-6) |
| *3.2, 3.3, 3.4* | *Training strategies: Describe the AI method/application used, with rationale for clinical task fit*  Pretrained CT-ViT encoder fine-tuned for classification; appropriate for volumetric imaging. | Methods – AI model (pp. 5-6) |
| *3.5* | *Solving clinical problems* |  |
| a | For segmentation tasks, describe architecture choice and temporal data handling | N/A |
| b | For NLP/report generation, indicate domain-specific fine-tuning and evaluation | N/A |
| c | If combining multiple data sources, provide rationale and integration method  CT imaging + echo report derived labels. No multimodal fusion at inference. | Methods – AI model + Baseline model (pp. 5-6) |
| **4** | **Model Assessment** |  |
| *4.1* | *Importance of model assessment: Describe the evaluation approach and how it addresses the clinical question*  Evaluation includes discrimination, calibration, subgroup analysis, and clinical utility. | Methods – Evaluation metrics (pp. 6-7), Results (pp. 9–13) |
| *4.2* | *Technical performance metrics* |  |
| a | Report relevant performance metrics (F1-score, IoU, BLEU, etc.)  AUROC, F1-score, balanced accuracy, Brier score, PR-AUC, decision-curve analysis. | Results – AI Model Evaluation (pp. 9–10), Fig. 4 |
| b | Justify metric selection based on task characteristics  Metrics chosen for classification, imbalance, and clinical calibration. | Methods – Evaluation metrics (pp. 6-7) |
| c | Describe manual annotation process, reference-standard prep, and observer variability  Echo-derived LVEF (2D echocardiography); inter-operator variability acknowledged. | Methods – Evaluation metrics (pp.6-7), Discussion (pp. 13-17) |
| *4.3, 4.4* | *Robustness, generalizability, and evaluating data quality* |  |
| a | Evaluate model robustness to external variations (scanners, protocols, institutions)  WCM dataset as external validation and independent consecutive test cohorts. | Results – External validation (p. 10) |
| b | Assess performance across clinical subpopulations and sociodemographic subgroups  Age, sex, race, and CT–echo time intervals. | Results – Subgroup analysis (p. 10), Fig. 4c |
| *4.5* | *Identifying features learned by the model* |  |
| a | Describe interpretability/explainability methods  Grad-CAM visualization. Radiologist manual examination and interpration. | Results – Model interpretation (pp. 11-12), Fig. 5 |
| b | Quantify and discuss uncertainty (epistemic, aleatoric)  Addressed via multi-seed variability (mean ± SD). | Methods – AI model (pp. 5-6), Results (pp. 9–10), Statistical Analysis (p. 17) |
| **5** | **Clinical Evaluation** |  |
| *5.1* | *Importance of clinical evaluation: Describe potential clinical impact of misclassification*  Potential early detection of asymptomatic HF; trade-offs discussed. | Discussion (pp. 14–15) |
| *5.2* | *Clinical utility metrics* |  |
| a | Define clinical utility of the AI system  Screening for abnormal EF to guide echocardiography referral. | Discussion (p. 14) |
| b | Describe cost-effectiveness analysis (throughput, resource utilization)  Discussed via NNE and downstream testing considerations. | Discussion (p. 14) |
| *5.3* | *Clinical validation: Provide evidence of clinical validation*  External validation + independent consecutive set validation. | Results – External + independent consecutive set validation (pp. 10, 12-13) |
| *5.4* | *Continuous monitoring: Outline plans for post-deployment monitoring*  Future prospective studies and deployment considerations discussed. | Discussion (pp. 14-15) |
| **6** | **Best Practices for Replicability** |  |
| *6.1* | *Importance of transparency and open science principles* |  |
| a | Ensure data sharing follows FAIR principles  Not publicly available (clinical data). | Discussion (p. 14) |
| b | Report on training data representativeness  Multi-center cohort with demographic diversity. | Results – Table 1 (pp. 27-28) |
| *6.2* | *Ensuring technical reproducibility* |  |
| a | Report results for the final model and training process  Reported as mean ± SD across 5 seeds. | Methods – AI model (pp. 5-6), Statistical Analysis (p. 17) |
| b | Describe sources of randomness in training  Random seed variation. | Methods – AI model (pp. 5-6) |
| c | Report random seeds (if applicable)  Five independent runs. | Methods – AI model (pp. 5-6) |
| d | Describe hardware setup  NVIDIA A100 GPU. | Methods – AI model (pp. 5-6) |
| e | Evaluate uncertainty from randomness (multiple training runs)  Quantified via multi-seed variability. | Results (pp. 10–13) |
| f | Justify if source code, model weights, or datasets are not shared  Code and model weights to be released upon publication. | Code and Model Availability (p. 18) |
| *6.3* | *Specific considerations for reproducing LLM and generative AI studies N/A* | N/A |
| a | Report model name, version, provider, and access date |  |
| b | Describe exact prompts and key generation parameters |  |
| c | Clarify usage context (API vs. local, fine-tuning) |  |
| d | Explain approach to handling output variability |  |
| e | Enable independent testing if full reproducibility is not possible |  |
| **7** | **Reporting of Limitations, Biases, and Alternatives** |  |
| *7.1* | *Acknowledging study and model limitations* |  |
| a | Discuss key limitations (data, methodology, generalizability)  Retrospective design, label noise, echo variability, lack of standardized EF measurement. | Discussion (pp. 15–16) |
| b | Report sensitivity analyses and model-specific issues  Time-interval stratification. | Results – time interval analysis (pp. 9–10) |
| *7.2* | *Discussing study strengths: Articulate strengths (methodological rigor, dataset quality, innovation, clinical relevance)*  Large multi-center dataset, external validation, clinical utility analysis, interpretability. | Discussion (pp. 13–17) |
| *7.3* | *Reporting on bias and fairness assessment* |  |
| a | Report stratified performance metrics for demographic subgroups  Stratified performance reported. | Results (pp. 9–10) |
| b | Describe bias mitigation strategies and residual bias  No explicit mitigation. | Discussion (pp. 15-16) |
| c | Acknowledge fairness evaluation limitations  Limitations acknowledged. | Discussion (pp. 15-16) |
| *7.4* | *Contextualizing with alternative methods* |  |
| a | Benchmark against clinical standards/traditional risk scores  Compared with radiologists and baseline RF model. | Results – Radiologist comparison (pp. 11-12) |
| b | Justify complex AI models over simpler alternatives  Deep learning justified over simpler models through numerical analyses. | Discussion (p. 15) |
| c | Discuss complementary methods (e.g., radiomics) for validation  Alternative approaches (e.g., radiomics) discussed conceptually. | Discussion (p. 15) |

**Supplementary Table 9. Compliance with PRIME 2.0 checklist** This table summarizes the compliance of the current study with the Proposed Requirements for Cardiovascular Imaging-Related Multimodal-AI Evaluation ([https://pubmed.ncbi.nlm.nih.gov/40892627/](https://nam12.safelinks.protection.outlook.com/?url=https%3A%2F%2Fpubmed.ncbi.nlm.nih.gov%2F40892627%2F&data=05%7C02%7Czib4001%40med.cornell.edu%7Cbf4126fee5864e60878208dea486230e%7Cf71b58b5412643a9bcb321c9de6f5241%7C0%7C0%7C639129094289113024%7CUnknown%7CTWFpbGZsb3d8eyJFbXB0eU1hcGkiOnRydWUsIlYiOiIwLjAuMDAwMCIsIlAiOiJXaW4zMiIsIkFOIjoiTWFpbCIsIldUIjoyfQ%3D%3D%7C0%7C%7C%7C&sdata=UHcK8%2FWjZ4782B53rrLmrUUSv7QtlACsF6JWZSJzLMs%3D&reserved=0)) (PRIME 2.0) checklist, with corresponding locations in the main manuscript and supplementary materials.
